# Kukri snakes *Oligodon* Fitzinger, 1826 of the Western Palearctic with the resurrection of *Contia transcaspica* Nikolsky, 1902 (Reptilia, Squamata, Colubridae)

**DOI:** 10.7717/peerj.15185

**Published:** 2023-05-18

**Authors:** Justin L. Lee, Platon V. Yushchenko, Konstantin D. Milto, Mahdi Rajabizadeh, Eskandar Rastegar Pouyani, Daniel Jablonski, Rafaqat Masroor, Suranjan Karunarathna, Ashok Kumar Mallik, Princia Dsouza, Nikolai Orlov, Roman Nazarov, Nikolay A. Poyarkov

**Affiliations:** 1 Department of Biology and Center for Biodiversity and Ecosystem Stewardship, Villanova University, Villanova, United States; 2 Department of Vertebrate Zoology, National Museum of Natural History, Smithsonian Institution, Washington, DC, United States; 3 Faculty of Biology, Department of Vertebrate Zoology, Moscow State University, Moscow, Russia; 4 Joint Russian-Vietnamese Tropical Research and Technological Center, Hanoi, Vietnam; 5 Zoological Institute, Russian Academy of Sciences, St. Petersburg, Russia; 6 Department of Biodiversity, Institute of Science and High Technology and Environmental Sciences, Graduate University of Advanced Technology, Kerman, Iran; 7 Hakim Sabzevari University, Sbazavar, Iran; 8 Department of Zoology, Comenius University in Bratislava, Bratislava, Slovak Republic; 9 Zoological Sciences Division, Pakistan Museum of Natural History, Islamabad, Pakistan; 10 Nature Explorations and Education Team, Moratuwa, Sri Lanka; 11 Center for Ecological Sciences, Indian Institute of Science, Bangalore, Karnataka, India; 12 Department of Wildlife and Biodiversity Conservation, Maharaja Sriram Chandra Bhanja Deo University, Baripada, Odisha, India; 13 Zoological Museum, Moscow State University, Moscow, Russia

**Keywords:** *Oligodon transcaspicus*, *Oligodon taeniolatus*, Middle Asia, Iran, Turkmenistan, Köpet–Dag Mountain Range, Indian subcontinent, Taxonomy, mtDNA, species distribution modeling

## Abstract

The kukri snakes of the genus *Oligodon* Fitzinger, 1826 reach the westernmost limits of their distribution in Middle and Southwest Asia (Afghanistan, Iran, and Turkmenistan), and the Palearctic portions of Pakistan. In this article, we review the systematics and distribution of the two species native to this region, *Oligodon arnensis* (Shaw, 1802) and *Oligodon taeniolatus* (Jerdon, 1853) based on an integrative approach combining morphological, molecular, and species distribution modeling (SDM) data. Phylogenetic analyses recover *O. taeniolatus* populations from Iran and Turkmenistan in a clade with the *O. arnensis* species complex, rendering the former species paraphyletic relative to *O. taeniolatus sensu* stricto on the Indian subcontinent. To correct this, we resurrect the name *Contia transcaspica* Nikolsky, 1902 from the synonymy of *O. taeniolatus* and assign it to populations in Middle–Southwest Asia. So far, *Oligodon transcaspicus* **comb. et stat. nov.** is known only from the Köpet–Dag Mountain Range of northeast Iran and southern Turkmenistan, but SDM mapping suggests it may have a wider range. Genetic samples of *O. “arnensis”* from northern Pakistan are nested in a clade sister to the recently described *Oligodon churahensis* Mirza, Bhardwaj & Patel, 2021, and are phylogenetically separate from *O. arnensis sensu* stricto in south India and Sri Lanka. Based on morphological similarity, the Afghanistan and Pakistan populations are assigned to *Oligodon russelius* (Daudin, 1803) and we synonymize *O.* *churahensis* with this species. Our investigation leads us to remove *O. taeniolatus* from the snake fauna of Afghanistan, Iran, and Turkmenistan, with the consequence that only *Oligodon transcaspicus* **comb. et stat. nov.** and *O. russelius* are present in these countries. Additional studies are needed to resolve the taxonomy of the *O. taeniolatus* and *O. arnensis* species complexes on the Indian subcontinent, and an updated key for both groups is provided.

## Introduction

Knowledge of the snake fauna of Middle and Southwest Asia (here considered the countries of Afghanistan, Iran, western China, Mongolia and the former Soviet Middle Asian republics fide Berg, 1931; Geptner, 1938; Chernov, 1949) has improved over the past decade as researchers continue to contribute species descriptions, range extensions, and natural history observations (Wagner et al., 2016a; Rajabizadeh, 2018; Shestopal & Rustamov, 2018a; Shestopal & Rustamov, 2018b; Orlov et al., 2018; Asadi et al., 2019; Rajabizadeh et al., 2020; Eskandarzadeh et al., 2020; Chen et al., 2021). Biogeographically, most snakes inhabiting Middle–Southwest Asia are elements of the Palearctic; however, a few species traditionally associated with the Indo-Malayan (Oriental) realm reach their westernmost distributional limits in this region (Wagner et al., 2016b; Orlov et al., 2018). The kukri snakes of the genus *Oligodon* Fitzinger, 1826 are one such example. Normally found across tropical portions of South and Southeast Asia, two species of this diverse colubrid radiation extend into Afghanistan, northeastern Iran, and southern Turkmenistan (Latifi, 1991; Green, 2010; Wagner et al., 2016b; Orlov et al., 2018; Bandara et al., 2022; Uetz et al., 2023).

The first species, the banded kukri snake *Oligodon arnensis* (Shaw, 1802), is common across the Indian subcontinent but a single specimen from Afghanistan is known (Král, 1969; Wagner et al., 2016b). This species was recently divided into three taxa by Bandara et al. (2022), who published a revision of *O. arnensis sensu* auctorum based largely on morphology. These authors restricted *O. arnensis sensu* stricto to southern India and Sri Lanka; moreover, they described a new species *Oligodon tillacki* Bandara et al., 2022 (Tillack’s kukri snake) for *O. arnensis* populations in western India, and resurrected the name *Oligodon russelius* (Daudin, 1803) (Russell’s kukri snake) for populations in northern/eastern India, Bangladesh (Barkat & Rabbe, 2022) and Nepal (Rai, Adhikari & Antón, 2022). Additionally, Mirza, Bhardwaj & Patel (2021) described another species related to *O. arnensis* from the western Himalayan foothills of India, *Oligodon churahensis* Mirza, Bhardwaj & Patel, 2021 (Churah Valley kukri snake). These latter authors included mitochondrial DNA (mtDNA) sequences of *O. “arnensis”* from Pakistan that were closely related to their new species (~3.3% genetic divergence based on the cytochrome *b* gene) but were unable to examine the vouchers corresponding to these samples and tentatively identified them as *O*. cf. *churahensis*. Although these studies have shed light on the taxonomic nature of *O. arnensis*, they both neglected the status of specimens from Pakistan and Afghanistan. We refer to these populations as *O. “arnensis”* until our clarifications in the results.

The second species, the streaked kukri snake *Oligodon taeniolatus* (Jerdon, 1853), also inhabits the Indian subcontinent, but westward localities are known from northern Iran, southern Turkmenistan, and possibly Afghanistan (Latifi, 1991; Wagner et al., 2016b; Orlov et al., 2018). Like *O. “arnensis”*, *O. taeniolatus* is phenotypically variable across its range and contains multiple color morphotypes that have been recognized as synonyms or subspecies by past authors (Wall, 1921; Wall, 1923; Smith, 1943; Taylor, 1950). The complicated nomenclatural history of *O. taeniolatus* was reviewed by Bauer (2003). In that review, Bauer (2003) designated a lectotype for the species based on an illustrated specimen depicted in Russell (1796) with the type locality “Vizagapatam” (now Visakhapatnam, Andhra Pradesh, India), thereby restricting ‘true’ (nominotypical) *O. taeniolatus* to eastern India. Outside of the Indian subcontinent, populations of *O. taeniolatus* in Iran and Turkmenistan can be referred to the name *Contia transcaspica* Nikolsky, 1902, described based on a single specimen collected from “Köpet–Dag, Transcaspia” (=now Köpet–Dag Mountain Range, near Ashgabat, Ahal Province, Turkmenistan). Chernov (1935) considered *C. transcaspica* to be a junior synonym of *O. taeniolatus*, owing to similarities between Turkmen and Indian specimens. Later authors would follow this decision and report *Oligodon* populations from Turkmenistan and neighboring Iran as *O. taeniolatus* (Terentjev & Chernov, 1949; Bogdanov, 1962; Bannikov et al., 1977; Dotsenko, 1984; Atayev, 1985; Szczerbak, Khomustenko & Golubev, 1986; Latifi, 1991; Rustamov & Sopyev, 1994; Szczerbak, 1994; Atayev, Rustamov & Shammakov, 1994). The name *Contia transcaspica* has rarely been mentioned since and was neglected in the synonymy of *O. taeniolatus* by Wallach, Williams & Boundy (2014). Since Chernov (1935), only a few sources (Dotsenko, 1984; Latifi, 1991; Latifi, 2000; Rajabizadeh, 2018) have provided additional descriptive data on *O. taeniolatus* from Iran and Turkmenistan. Though their accounts do not make direct comparisons with Indian material, it is apparent that specimens from Middle and Southwest Asia do not accord well with the traditional definition of *O. taeniolatus* (Wall, 1921; Wall, 1923; Smith, 1943).

In this study, we review the taxonomy, phylogenetic relationships, and distributional limits of the genus *Oligodon* at the westernmost end of its range. Our geographic focus includes all Middle and Southwest Asian countries inhabited by kukri snakes, in addition to Pakistan, which is traditionally part of South Asia but still encompasses part of the western Palearctic and contains the same species treated here. Chiefly, we aim to clarify the status of *O. taeniolatus* populations historically associated with the name *Contia transcaspica* from Iran and Turkmenistan and resolve the status of *Oligodon “arnensis”* populations from Pakistan and Afghanistan, which were neglected by the latest taxonomic revisors.

## Materials and Methods

### Sampling and species delimitation

**Figure 1:**
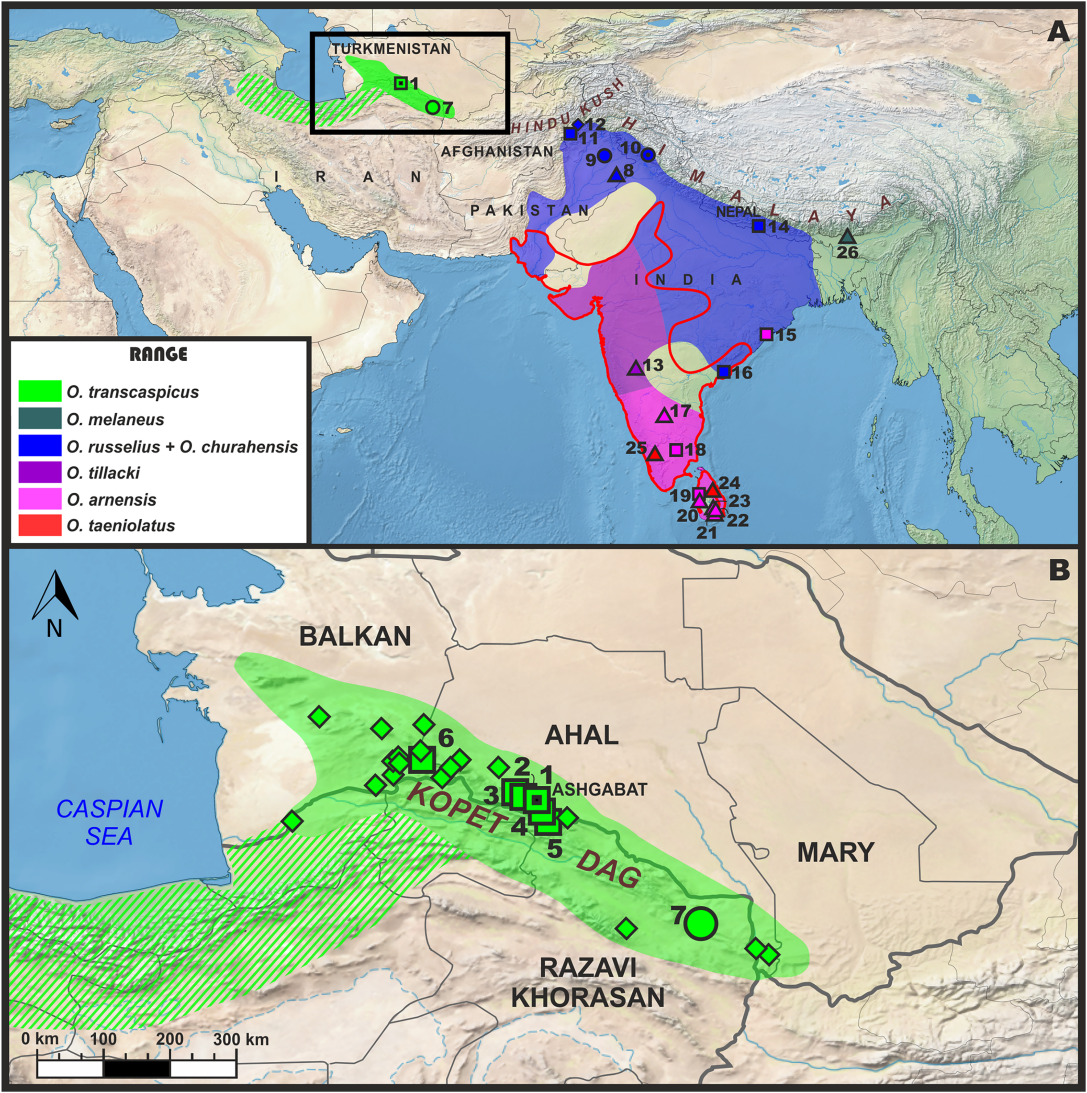
Approximate distribution and location of samples utilized in this study for molecular and morphological analyses (A); and approximate distribution of *Oligodon transcaspicus* comb. et stat. nov in the Köpet–Dag Mountain range of Iran and Turkmenistan. Triangle icons denote specimens with only molecular data; square icons denote specimens with only morphological data; rhombic icons denote specimens without molecular and morphological data (only locality is known); round icons denote specimens with both molecular and morphological data. Stars/asterisks next to numbers indicate type localities. Localities: (1) Ashgabat, Ahal Prov., Turkmenistan (type locality of *Oligodon transcaspicus* **comb. et stat. nov**); (2) Chuli, Ahal Prov., Turkmenistan; (3) Geok-Tepe, Ahal Prov., Turkmenistan; (4) Karanki, Ahal Prov., Turkmenistan; (5) “Goalon” (Goudan), Ahal Prov., Turkmenistan; (6) Aidere, Ahal Prov., Turkmenistan; (7) Bazangan Lake, Razavi Khorasan Prov., Iran; (8) Punjab Prov., Pakistan; (9) Kallar Kahar, Punjab Prov., Pakistan; (10) Chamba Distr., Himachal Pradesh, India (type locality of *Oligodon churahensis*); (11) Jalalabad, Nangarhar Prov., Afghanistan; (12) Dara-i-Pech district, Kunar Prov., Afghanistan; (13) Maharashtra, India; (14) Chitwan NP, Bagmati Prov., Nepal; (15) Ganjam, Odisha, India; (16) 24.1 km SW of Rajamahendravaram, Andhra Pradesh, India; (17) Bangalore, Karnataka, India; (18) Tamil Nadu, India; (19) Puttalam Distr., North Western Prov., Sri Lanka; (20) Udawalawe National Park, Uva Prov.; (21) Monargala Distr., Southern Prov., Sri Lanka; (22) Hambantota Distr., Southern Prov., Sri Lanka; (23) Gampaha Distr., Western Prov., Sri Lanka; (24) Polonnaruwa Distr., North Central Prov., Sri Lanka; (25) Paramikulam, Kerala, India; (26) Barengabari, Assam, India.

Fieldwork which resulted in the collection of *Oligodon* in Iran was conducted by RN and MR during a field trip in May 2017 to Razavi, Khorasan Province, Iran (locality 7, Fig. 1). Fieldwork in Pakistan was conducted by DJ and RM during a field trip in September 2018 to Punjab Province, Pakistan. Fieldwork in Sri Lanka was conducted by SK and NAP during field trips between February 2018 and December 2020 to the dry and wet zones of the country. Collected specimens were euthanized using a 20% solution of benzocaine and fixed in formalin before being transferred into 70% ethanol for storage. Before preservation, a small sample of muscle tissue was taken from each snake and stored in 95% ethanol for subsampling and DNA extraction. For molecular phylogenetic analyses, we included one sample of *O. taeniolatus* (referrable to *Contia transcaspica*) from Iran (ZMMU Re-16687); one sample of nominotypical *O. taeniolatus* from India (CESS-180); four novel samples of *O. arnensis sensu* stricto from India (CESS-563) and Sri Lanka (SL-Os-1, SL-Oa-2; ZMMU Re-17331); two novel samples of *O. sublineatus* Duméril, Bibron & Duméril, 1854 from Sri Lanka (SL-Os-2; SL-Os-3); and one novel sample morphologically resembling *Oligodon russelius* from Punjab Province, Pakistan (CUHC 7904; locality 9, Fig. 1). In addition, we included 53 publicly available sequences of other *Oligodon* species retrieved from GenBank (Table 1). Bandara et al. (2022) noted that one sample (NCBS-NRC-AA-021) previously identified as *O. “arnensis”* may represent *O. tillacki* but did not examine this specimen. As such, we re-identify this specimen as *O*. cf. *tillacki* herein (see Discussion). For outgroup taxa, we chose one sample each of *Oreocryptophis porphyraceus* (Cantor, 1839) (subfamily Colubrinae) and *Hebius vibakari* (Boie, 1826) (subfamily Natricinae) due to their use in previous phylogenies of *Oligodon* published in the literature (Nguyen et al., 2020; Yushchenko et al., 2023a, 2023b). One specimen (ZMMU Re-16687) will be transferred to the International Center for Science, High Technology and Environmental Sciences Herpetological Collection (ICSTZM) in order to meet the obligations of fieldwork permissions.

**Table 1:** Sequences and voucher specimens of *Oligodon* and outgroup taxa used in molecular analyses of this study.

No.	Sample ID	GenBank accession no.	*Species*	Country	Locality	Reference
1	ZMMU Re-16687	OQ116823; OQ116816	*Oligodon transcaspicus* **comb. et stat. nov.**	Iran	Razavi Khorasan Prov., Bazangan Lake	This work
2	CUHC 7904	OQ092426; OQ116817	*Oligodon russelius*	Pakistan	Punjab Prov., Kallar Kahar	This work
3	SL-Os-1	OQ099833; OQ116819	*Oligodon arnensis albiventer*	Sri Lanka	Western Prov., Gampaha Distr., Ganemulla	This work
4	SL-Oa-2	OQ116825; OQ116820	*Oligodon arnensis albiventer*	Sri Lanka	Sabaragamu Prov., Ratnapura Distr., Udawalawe	This work
5	ZMMU Re-17331	OQ116824; OQ116818	*Oligodon arnensis albiventer*	Sri Lanka	Uva Prov., Monargala Distr., Thanamalwila	This work
6	SL-Os-2	OQ099834; OQ116821	*Oligodon sublineatus*	Sri Lanka	Central Prov.	This work
7	SL-Os-3	OQ099835; OQ116822	*Oligodon sublineatus*	Sri Lanka	Central Prov., Kandy Distr.	This work
8	CESS 563	OQ099837	*Oligodon arnensis*	India	Karnataka, Bangalore	This work
9	CESS 180	OQ099836	*Oligodon taeniolatus*	India	Kerala, Paramikulam	This work
10	WII-ADR980	ON262767; ON241309	*Oligodon melaneus*	India	Assam, Barengabari	Das et al. (2022)
11	NCBS NRC-AA-019	MZ675817	*Oligodon churahensis*	India	Himachal Pradesh, Chamba Distr.	Mirza, Bhardwaj & Patel (2021)
12	NCBS NRC-AA-020	MZ675818	*Oligodon churahensis*	India	Himachal Pradesh, Chamba Distr.	Mirza, Bhardwaj & Patel (2021)
13	ZMUVAS 10	MK941834	*Oligodon russelius*	Pakistan	Punjab Prov.	Mirza, Bhardwaj & Patel (2021)
14	Saeed 5	MZ403752	*Oligodon russelius*	Pakistan	–	S. Ahmed et al. (2021, unpublished)
15	RAP 483	KC347327; KC347365; KC347464	*Oligodon arnensis*	Sri Lanka	Southern Prov., Hambantota Distr.	Pyron et al. (2013)
16	NCBS-NRC-AA-021	MZ675819	*Oligodon cf. tillacki*	India	Maharashtra	Mirza, Bhardwaj & Patel (2021)
17	RS136	KC347330; KC347368; KC347484; KC347521; KC347408; KC347445	*Oligodon taeniolatus ceylonicus*	Sri Lanka	Central Prov., Polonnaruwa Distr.	Pyron et al. (2013)
18	RS-OC	KC347328; KC347366	*Oligodon calamarius*	Sri Lanka	Central Prov., Kandy Distr.	Pyron et al. (2013)
19	RAP 504	KC347329; KC347367	*Oligodon sublineatus*	Sri Lanka	Central Prov., Kandy Distr.	Pyron et al. (2013)
20	ROM37092	HM591504	*Oligodon cinereus*	Vietnam	Dong Nai Prov., Cat Tien NP	Green, Orlov & Murphy (2010)
21	UMMZ201913	HM591519	*Oligodon octolineatus*	Brunei	Tutong Distr., 3 km E of Tutong	Green, Orlov & Murphy (2010)
22	ROM 35626	HM591526	*Oligodon chinensis*	Vietnam	Cao Bang Prov., Quang Thanh	Green, Orlov & Murphy (2010)
23	ROM35629	HM591533	*Oligodon formosanus*	Vietnam	Cao Bang Prov., Quang Thanh	Green, Orlov & Murphy (2010)
24	ROM32261	HM591534	*Oligodon ocellatus*	Vietnam	Dak Lak Prov., Yok Don NP	Green, Orlov & Murphy (2010)
25	ROM32260	HM591521	*Oligodon taeniatus*	Vietnam	Dak Lak Prov., Yok Don NP	Green, Orlov & Murphy (2010)
26	ROM32464	HM591523	*Oligodon barroni*	Vietnam	Gia Lai Prov., Krong Pa	Green, Orlov & Murphy (2010)
27	CAS204963	HM591535	*Oligodon cyclurus*	Myanmar	Ayeyarwady Reg., Mwe Hauk	Green, Orlov & Murphy (2010)
28	CAS204855	HM591509	*Oligodon splendidus*	Myanmar	Mandalay Reg., Kyauk Se	Green, Orlov & Murphy (2010)
29	CAS215976	HM591513	*Oligodon torquatus*	Myanmar	Mandalay Reg., Min Gone Taung WS	Green, Orlov & Murphy (2010)
30	CAS213822	HM591514	*Oligodon planiceps*	Myanmar	Magwe Reg., Shwe Set Taw WS	Green, Orlov & Murphy (2010)
31	CAS213896	HM591516	*Oligodon theobaldi*	Myanmar	Magwe Reg., Shwe Set Taw WS	Green, Orlov & Murphy (2010)
32	CAS213271	HM591517	*Oligodon cruentatus*	Myanmar	Yangon Reg., Hlaw Ga NP	Green, Orlov & Murphy (2010)
33	ROM27049	HM591518	*Oligodon eberhardti*	Vietnam	Cao Bang Prov., Quang Thanh	Green, Orlov & Murphy (2010)
34	TNHC59846	HM591511	*Oligodon maculatus*	Philippines	Mindanao, Barangay Baracatan	Green, Orlov & Murphy (2010)
35	SIEZC 20201	MN395604; MN396765	*Oligodon rostralis*	Vietnam	Lam Dong Prov., Bidoup–Nui Ba NP	Nguyen et al. (2020)
36	ZMMU Re-14304	MN395601; MN396762	*Oligodon annamensis*	Vietnam	Dak Lak Prov., Chu Yang Sin NP	Nguyen et al. (2020)
37	KIZ014591	MW090140; MW133297	*Oligodon nagao*	–	–	Xu et al. (2021)
38	KIZ011002	MW090139; MW133296	*Oligodon lipipengi*	China	Tibet, Medok	Xu et al. (2021)
39	CHS850	MK194265; MK201568; MK065694	*Oligodon albocinctus*	China	–	J. Li et al. (2021, unpublished)
40	CHS668	MK194135; MK201461; MK065563	*Oligodon fasciolatus*	China	–	J. Li et al. (2021, unpublished)
41	CHS304	MK194038; MK201386; MK065470	*Oligodon lacroixi*	China	–	J. Li et al. (2021, unpublished)
42	CHS683	MK194147; MK065575	*Oligodon ornatus*	China	–	J. Li et al. (2021, unpublished)
43	SYNU 1907027	MW489824	*Oligodon bivirgatus*	China	Hainan, Shangxi NR	Qian et al. (2021)
Outgroups						
44	–	KP684155	*Hebius vibakari*	–	–	–
45	–	GQ181130	*Oreocryptophis poryphyraceus*	–	–	–

**Note:**

Note that the numbers (column one) included in this table are not the same as those found in other tables or figures. Acronyms present here that are not noted in the materials and methods include the following: CHS, unknown field tag series; KIZ, Kunming Institute of Zoology, Chinese Academy of Sciences, Kunming, China; NCBS, National Center for Biological Sciences, Bangalore, India; RAP, field tags of R. Alexander Pyron; ROM, Royal Ontario Museum, Toronto, Canada; RS, field tags of Ruchira Somaweera; SIEZC, Department of Zoology, Southern Institute of Ecology, Ho Chi Minh City, Vietnam; SYNU, Shenyang Normal University, Shenyang, China; TNHC, Texas Natural History Collections, Austin, USA; UMMZ, University of Michigan Museum of Zoology, Ann Arbor, USA; ZMUVAS, Zoological Museum of the University of Veterinary and Animal Sciences, Punjab, Pakistan. Additional abbreviations include NP, national park; NR, nature reserve; WS, wildlife sanctuary.

We examined the morphology of seven *O. taeniolatus* specimens available to us from Iran and Turkmenistan, including the name-bearing type of *Contia transcaspica* (localities 1–7, Fig. 1). To compare these populations with typical members of the *O. arnensis* species complex (*sensu* Bandara et al., 2022), we examined two specimens within the range of *O. russelius* from Andhra Pradesh, India, and southern Nepal (localities 14–16, Fig. 1), five specimens of *O. arnensis sensu* stricto from southern India and Sri Lanka (localities 18–19, Fig. 1) (see Material S1), and a new specimen of *O. russelius* collected from Pakistan (locality 9, Fig. 1). Additional morphological data for *O. taeniolatus*, the *O. arnensis* species complex, and other taxa were derived from relevant literature sources (see Morphological analyses). For aspects of species concepts and delimitation, we act in accordance with an integrative taxonomic approach (Padial et al., 2010), where the delimitation of a species is supported by a combination of morphological, molecular, and ecological evidence. Furthermore, we follow the General Lineage Concept (De Queiroz, 2007), where a species represents a single independent lineage following a separate evolutionary trajectory compared to its congeners. Criteria we used to support the existence of evolutionary independence include discrete morphological separation, substantial genetic divergence based on standard genetic markers, and evidence of monophyly when phylogenetically compared with other congeners.

The following acronyms for museum and natural history collections are used in the text: CAS: California Academy of Sciences, San Francisco, USA; CUHC: Comenius University Herpetological Collection, Bratislava, Slovakia; MMB: Department of Zoology, Moravian Museum, Brno, Czech Republic; USNM: National Museum of Natural History, Washington, DC, USA; ZISP: Zoological Institute of Russian Academy of Sciences, St. Petersburg, Russia; ZMMU: Zoological Museum of Moscow State University, Moscow, Russia. Additional abbreviations for genetic samples and voucher specimens can be found in Table 1.

Permission to conduct fieldwork in Iran, including collection of samples and animals in the field, was performed outside of any protected area, in the framework of a project contract signed by International Center for Science, High Technology and Environmental Sciences, Kerman, Iran (contract number 1.87, issued 11.04.2008). This contract bears permission to collect reptile samples outside of any protected area of the Department of the Environment (specified in www.doe.ir) that require extra permissions. Specimen collection protocols and animal operations followed the Institutional Ethical Committee of International Center for Science, High Technology and Environmental Sciences, Kerman, Iran (certificate number 1.87-1). Fieldwork to collect specimens in Pakistan was conducted under the permit of the Pakistan Museum of Natural History, Islamabad, Pakistan (No. PMNH/EST-1[89]/05). In Sri Lanka, permission to conduct fieldwork was granted by the Department of Wildlife Conservation and Department of Forest Conservation (WL/3/2/1/14/12).

### Molecular analyses

Methods for DNA sequencing and molecular phylogenetic analyses are adapted from previous studies (Yushchenko et al., 2023a, 2023b). We extracted total genomic DNA of novel samples from muscle tissue preserved in 95% ethanol using a Qiagen DNAeasy Blood and Tissue Kit following manufacturer protocols. We performed polymerase chain reactions (PCRs) on extracted DNA to amplify two fragments of mitochondrial DNA (mtDNA): the first fragment including partial sequences of 12S ribosomal RNA (rRNA), transfer RNA (tRNA)-Valine and 16S rRNA (total length up to 1,930 bp), and a second fragment including the complete sequence of the gene Cytochrome *b* (cyt *b*) (1,091 bp). Primers used for PCRs and sequencing are summarized in Table S2. PCR protocols for 12S–16S rRNA fragments were adapted from Green, Orlov & Murphy (2010). For both primer pairs of 12S and 16S rRNA, we used the following PCR protocols: (1) initial denaturation step at 94 °C for 5 min; (2) 35 cycles of denaturation at 94 °C for 1 min, annealing at 55 °C for 1 min and extension at 72 °C for 1 min; (3) final extension at 72 °C for 10 min; and (4) cooling step at 4 °C for storage. For cyt *b* sequences, we used a modified PCR protocol of Chen et al. (2014) with touchdown: (1) initial denaturation step at 94 °C for 5 min; (2) 10 cycles of denaturation at 94 °C for 1 min, annealing for 1 min with temperature decreasing from 50 °C to 45 °C (with cool-down at 0.5 °C per each cycle) and extension at 72 °C for 1 min; (3) 24 cycles of denaturation at 94 °C for 1 min, annealing at 45 °C for 1 min and extension at 72 °C for 1 min; (4) final extension at 72 °C for 10 min; and (5) cooling step at 4 °C for storage. All PCR products were sequenced in both directions by the Evrogen company at the Institute of Bioorganic Chemistry, Russian Academy of Sciences (Moscow, Russia) and at Macrogen Europe (Amsterdam, The Netherlands; http://www.macrogen-europe.com). Sequences were assembled and checked using Sequencher 4.9 (GeneCodes). The obtained sequences are deposited in GenBank under the accession numbers OQ092426; OQ099833–OQ099837; and OQ116816–OQ116825 (Table 1).

To examine the position of *O. taeniolatus* from Turkmenistan and Iran in a matrilineal genealogy of the genus, we combined newly obtained sequences with all publicly available GenBank sequences of *O. arnensis*, *O. churahensis*, *O. taeniolatus*, *O*. cf. *tillacki* and one sequence per species of other *Oligodon* (summarized in Table 1). The morphological identities of some samples remain unverified due to a lack of voucher material. In total, we analyzed mtDNA sequences of 45 specimens, including 43 samples of 33 species of *Oligodon*, with outgroup sequences of *Oreocryptophis porphyraceus* and *H. vibakari* from GenBank used to root the tree. Nucleotide sequences were initially aligned in MAFFT v.7 (Katoh & Standley, 2013) with default parameters, and subsequently checked by eye in BioEdit 7.0.5.2 (Hall, 1999) and slightly adjusted for translation when appropriate. We used IQ-TREE web server (http://iqtree.cibiv.univie.ac.at/; Trifinopoulos et al., 2016) to estimate optimal evolutionary models for the data set analysis using the Akaike Information Criterion (AIC). Mean uncorrected genetic distances (p-distances) were calculated in MEGA 7.0 (Kumar, Stecher & Tamura, 2016). The matrilineal genealogy was inferred using Bayesian inference (BI) and maximum likelihood (ML) approaches. The best-fitting model for both BI and ML analyses for 12S–16S rRNA fragments and for the second codon partition of cyt *b* was the GTR+G+I model as of DNA evolution suggested by the AIC. For the remaining portions of cyt *b*, the AIC suggested the GTR+G model for the first codon partition, and the HKY+G+I for the third codon partition.

The ML analysis was conducted using the IQ-TREE web server (Trifinopoulos et al., 2016). BI analysis was conducted in MrBayes 3.2.2 (Ronquist et al., 2012). For the BI analysis, Metropolis-coupled Markov chain Monte Carlo (MCMCMC) analyses were ran with one cold chain and three heated chains for one million generations and sampled every 1,000 generations. Two independent MCMCMC run iterations were performed and 100 trees were discarded as burn-in. The convergence of the runs was checked by exploring and examining likelihood plots in TRACER v1.6 (Rambaut et al., 2020), with effective sample sizes (ESS) all above 200. Nodal support for the BI analysis was assessed by calculating posterior probabilities (BI PP). We *a priori* regarded tree nodes with BI PP values over 0.95 as sufficiently resolved, whereas BI PP values between 0.95 and 0.90 were regarded as tendencies. For ML analysis, confidence in nodal topology was estimated *via* the ultrafast bootstrap approximation algorithm (UFBS; Hoang et al., 2018) with 1,000 bootstrap pseudoreplicates. Nodes having ML UFBS values of 95 and above were *a priori* considered highly supported, nodes with values of 90–94 were considered well-supported, and nodes with values of 70–89 were considered as tendencies. Lower values were regarded as indicating unresolved nodes (Huelsenbeck & Hillis, 1993).

### Morphological analysis

Coloration and pattern were recorded during examination of preserved specimens. For some specimens, live coloration was also recorded from digital images taken before preservation. No statistical analyses were performed between species due to the low sample size of comparative material. All body measurements, except body and tail lengths, were taken under a dissecting microscope using a digital slide-caliper to the nearest 0.1 mm. Body and tail lengths were measured to the nearest millimeter by straightening snakes along a flexible ruler. Methodology for counting ventral and subcaudal scales follow Dowling (1951). The tail tip was not included in the number of subcaudals. Head scale suture angle protocols follows that of Kaiser, O’Shea & Kaiser (2019). Maxillary teeth were counted by examination of the dissected maxillary bone when available, or by carefully removing the gum layer of the maxilla. Sex was determined by ventral incision below the vent to detect the presence or absence of hemipenes. Our data on specimens referrable to *Contia transcaspica* were compared and reviewed with relevant literature on *O. taeniolatus* across its distribution (Nikolsky, 1902; Nikolsky, 1903a; Wall, 1921; Wall, 1923; Chernov, 1935; Smith, 1943; Minton, 1966; Dotsenko, 1984; Latifi, 1991; Khan, 2002).

Linear morphometric data of examined specimens were collected as previously described by Nguyen et al. (2020) and Yushchenko et al. (2023a, 2023b). Specifically, the following linear measurements (all in mm) were taken: snout to vent length (SVL)—measured from the tip of the snout to the vent; tail length (TailL)—measured from the vent to the tip of the tail; total length (TotalL)—sum of SVL and TailL; relative tail length to total length (TailLR) calculated as tail length to total length ratio; head length (HeadL) from the tip of the snout to the posterior margin of the mandible; head width (HeadW) measured at the widest part of the head immediately posterior to the eye; snout length (SnoutL)—distance between the tip of the snout and anterior edge of eye; eye diameter (EyeD)—maximal horizontal length of the eye; frontal scale length (FrontalL); frontal scale width (FrontalW); distance (IOD)—the shortest distance between the eyes; and internarial distance (IND)—distance between the nostrils. Morphological characters examined also follow past descriptions of Yushchenko et al. (2023a, 2023b), and include the number of maxillary teeth (MT); anterior scale rows (ASR)—namely number of scale rows at one head length behind the head; midbody scale rows (MSR)—the number of scale rows at midbody (namely halfway between the measured snout-vent length); posterior scale rows (PSR)—number of dorsal scale rows at one head length prior to the vent; dorsal scale row formula (DSR)—a given acronym summarizing the three dorsal scale row counts (*i.e*., ASR–MSR–PSR); ventral scales (VEN)—the number of ventral scales starting from the scale contacting the first dorsal scale row to the vent, excluding the cloacal plate; subcaudal scales (SC)—the number of paired subcaudal scales excluding the terminal scute; total body scales (TOTAL)—the sum of ventral, subcaudal scales and the cloacal plate (included as one scale regardless of whether the plate is single or divided); subcaudal ratio (SCR)—namely the ratio between the number of subcaudal scales and the number of total body scales given as a decimal value; cloacal plate (CP)—the number of terminal ventral scales immediately anterior to vent (given as single for one scale, and divided for two scales); condition of nasal scale (NASAL)—given as either vertically divided, entire, or partially divided; condition of loreal scale (LOREAL)—given as present or absent; supralabials (SL)—the number of scales on upper lip; number of supralabials in contact with the eye (SL-Eye); infralabials (IL)—the number of scales on lower lip; infralabials contacting each other (IL-contact)—the number of pairs of infralabial scales in contact; infralabials contacting the anterior chin shields (IL-CS)—the number of infralabial scales contacting the anterior chin shields; number of preocular scales (PrO); number of presubocular scales (PrsO); number of postocular scales (PtO); number of anterior temporals (Ate)—the number of temporal scales in contact with the postocular scales; number of posterior temporals (Pte)—the number of temporal scales immediately contacting the anterior temporal scales. Besides these data, we quantified color pattern features found in examined *Oligodon*, including the number of body blotches or crossbars counted from nape to vent (B-Blotch); the number of blotches or crossbars counted from vent to tail tip (T-Blotch); the width in vertebral (dorsal) scales of each blotch/crossbar at midbody (BlotchW); and the distance between each blotch/crossbar at midbody counted using vertebral (dorsal) scales (BlotchD). Incomplete blotches/crossbars were counted as a single element, and partially fused blotches were counted as two elements. If a blotch/crossbar overlapped with the cloacal plate, it was counted as part of the number of body blotches. Abbreviations for these characters are used in Tables 2–4. Symmetric characters are given in left/right order.

**Table 2:** Selected morphological characters of *Oligodon transcaspicus* comb. et stat. nov. based on examined specimens.

Morphology	ZISP 9868 (holotype)	ZMMU Re-7318	ZMMU Re-5589	ZMMU Re-6155	CAS 180042	ZISP 18334	ZISP 18976	ZMMU Re-16687
Sex	f	f	f	f	f	m	m	m
SVL	304	361	267	166	338	145	252	312
TailL	53	64	42	29	64	27	53	70
TotalL	357	425	309	195	402	172	305	382
TailLR	0.148	0.151	0.136	0.149	0.159	0.157	0.174	0.183
DSR	15–15–15	15–15–15	15–15–15	15–15–15	15–15–15	15–15–15	15–15–15	15–15–15
VEN	202	198	203	193	214	188	188	179
SC	47	45	44	46	51	48	51	52
TOTAL	250	244	248	240	266	237	240	232
SCR	0.188	0.184	0.177	0.192	0.192	0.203	0.213	0.224
LOREAL	Present	Present	Present	Present	Present	Present	Present	Present
SL	5/5	5/5	5/5	5/5	5/5	5/5	5/5	5/5
SL-eye	3/3	3/3	3/3	3/3	3/3	3/3	3/3	3/3
IL	7/7	7/7	7/7	7/7	7/7	7/7	7/7	7/7
IL-CS	4/4	4/4	4/4	4/4	4/4	4/4	4/4	4/4
PrO	1/1	1/1	1/1	1/1	1/1	1/1	1/1	1/1
PsO	1/1	1/1	0/0	0/0	0/0	0/0	1/1	1/1
PtO	2/2	2/2	2/2	2/2	2/2	2/2	2/2	2/2
Ate	1/1	1/1	1/1	1/1	1/1	1/1	1/1	1/1
Pte	3/3	3/3	3/3	3/3	2/2	3/3	3/3	3/3
B-Blotch	47	52	57	54	54	47	43	42
T-Blotch	12	16	15	14	15	17	17	14

**Note:**

Abbreviations for males and females are denoted by (m) or (f) respectively. For abbreviations of characters, see materials and methods section for details.

**Table 3:** Morphological comparisons of *Oligodon russelius* specimens from Afghanistan, northern India, and Pakistan hitherto recognized as *Oligodon* “*arnensis*” or *Oligodon churahensis*.

Morphology	CUHC 7904	MMB 28497	*O. churahensis*	*O. russelius*	Pakistan *O. “arnensis”*	West Himalayan *O. “arnensis”*
Location	Kallar Kahar, Punjab Province, Pakistan	Jalalabad, Nangarhar, Afghanistan	Churah Valley, Himachal Pradesh, India	Northern and Eastern India	Northern and Southern Pakistan	Punjab and Himachal Pradesh, India
TailLR	0.159	0.136	0.180	0.157–0.185	0.160–175	0.163
VEN	183 (m)	198 (juv)	170 (m) 175 (f)	169–180 (m) 183–207 (f)	175–191 (m&f)	187–190* (m & f)
SC	49 (m)	44 (juv)	46 (m) 47 (f)	46–54 (m) 49–51 (f)	47–52 (m) 40 (f)	39–52* (m & f)
LOREAL	Present	Present	Present	Present	Present (rarely absent)	Present
SL	7	7	7	7	7	6–7
IL	7	6	7	7	7–8	7
B-Blotch	37	49 (total)	37–45	30–45	31–42	41–54
T-Blotch	13	—	9–11	6–10	—	9–13
Blotch edges	Present	—	Present	Mostly present	Present	Present
BlotchW	1.0–1.5	—	1.0–2.0	1.0–2.0	—	—
BlotchD	4.0	—	3.0–4.0	4.0–6.0	—	—
Source	This study	Brück (1968)	Mirza, Bhardwaj & Patel (2021)	Bandara et al. (2022)	Minton (1966), Mertens (1969), Khan (2002)	Wall (1921), Constable (1949)*

**Note:**

Data from the literature are combined from multiple authors unless denoted with an asterisk (*), which indicates that only a single source was used. Abbreviations for males and females are denoted by (m) or (f) respectively and (total) is used to indicate that the data combines both sexes or combines the total number of body and tail blotches.

**Table 4:** Pairwise matrix of genetic distances between and within *Oligodon* species sampled in this study.

No.	Species	1	2	3	4	5	6	7	8	9	10	11	12	13	14	15
1	*O. transcaspicus*	—														
2	*O. russelius*	6.3	**2.2**													
3	*O. arnensis*	6.9	6.1	**0.0**												
4	*O. melaneus*	7.8	6.4	6.3	—											
5	*O. sublineatus*	18.9	20.9	19.8	20.1	**0.0**										
6	*O. taeniolatus*	17.1	18.5	18.3	18.3	19.5	—									
7	*O. churahensis*	5.7	3.3	5.1	5.1	21.0	18.0	**0.0**								
8	*O. tillacki*	8.7	7.9	3.6	7.5	18.6	20.1	6.3	—							
9	*O. calamarius*	17.4	19.3	17.4	17.7	11.4	17.4	19.2	18.3	—						
10	*O. annamensis*	18.0	17.9	16.8	18.3	17.7	16.5	16.8	16.2	16.5	—					
11	*O. rostralis*	17.7	19.1	17.4	18.3	18.9	17.1	17.7	16.8	17.7	6.9	—				
12	*O. octolineatus*	16.8	16.7	16.2	16.2	16.5	16.2	15.9	15.9	16.8	12.9	12.0	—			
13	*O. albocinctus*	18.0	18.9	18.3	20.1	16.5	20.1	18.6	18.6	16.8	16.2	16.2	15.0	—		
14	*O. fasciolatus*	17.1	17.3	18.0	16.8	16.5	17.4	16.5	17.7	16.8	12.3	12.6	11.4	15.0	—	
15	*O. lacroixi*	16.2	16.8	15.6	17.4	16.8	16.8	16.2	15.6	15.3	13.2	15.0	11.7	15.9	15.6	—

**Note:**

Uncorrected p-distances (given as percentages) are based on sequences of the cytochrome *b* mtDNA gene are shown below the diagonal. Intraspecific genetic p-distances are shown along the diagonal and are highlighted in bold. See Table 1 for the list of samples used to create this matrix.

### Species distribution modeling (SDM)

We used the program MaxEnt 3.3.3 (Phillips, Anderson & Schapire, 2006) to model the potential distribution of *O. taeniolatus* populations in Middle and Southwest Asia during current conditions and three previous historical epochs, namely the mid-Pliocene (ca 3.2 Mya), the Last Glacial Maximum (ca 21 Kya), and the mid-Holocene (ca 6 Kya). A total of 23 unique georeferenced data points (Table S1), 23 bioclimatic variables, and four landscape layers were used to generate the model. We used the CHELSA database (Karger et al., 2017) to obtain current climatic conditions, and the PaleoCLIM database for past conditions during the mid-Pliocene, the last glacial maximum (LGM) and the Mid-Holocene (Fordham et al., 2017; Brown et al., 2018), at 5 km pixel size. The program ENMTools 1.3 (Warren, Glor & Turelli, 2010) was used to filter SDM data and exclude correlated variables. Models were assessed by computing the area under the curve (AUC), which was used to estimate the relative contribution of variables for each model. The final maps were designed using the QGIS Desktop 3.28 software (QGIS Development Team, 2021).

## Results

### Molecular analyses

The final concatenated alignment comprised of 12S–16S rRNA and cyt *b* gene sequences contained 3,019 base pairs with 1,812 conserved sites, 1,191 variable sites, and 833 parsimony informative sites. The transition-transversion bias (R) was estimated to be 1.2. Nucleotide frequencies were 36.7% (A), 23.0% (T), 25.7% (C), and 14.6% (G) (all data given for ingroups only). Uncorrected pairwise genetic distances (hereafter p-distances; given for cyt *b*) between and within examined *Oligodon* species are presented in Table 4. Intraspecific genetic distances varied from *p* = 0% (in *O. sublineatus*) to *p* = 2.2% (*O. churahensis*, including samples of *O*. cf. *churahensis* and *O. russelius*). Interspecific genetic distances within examined *Oligodon* varied from *p* = 3.3% (between *O. arnensis sensu* stricto and *O. churahensis*) to *p* = 21.0% (between *O. sublineatus* and *O. churahensis*).

**Figure 2:**
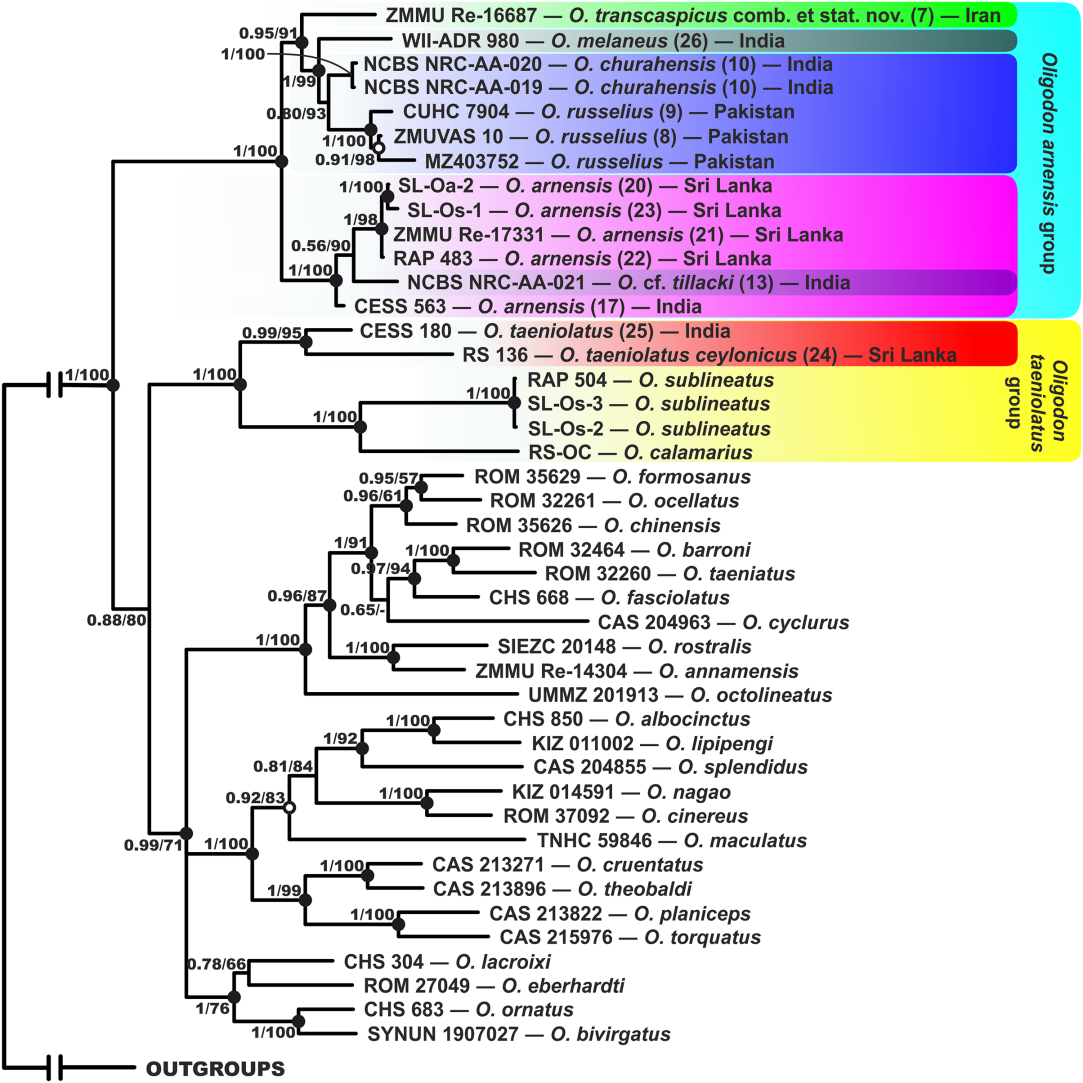
Phylogenetic tree of *Oligodon* derived from the analysis of 3,019 bp of 12S rRNA, 16S rRNA and cyt *b* mitochondrial DNA gene sequences. For voucher specimen information and GenBank accession numbers see Table 1. Numbers at tree nodes correspond to BI PP/ML BS support values, respectively; n-dash denotes no support. Outgroup taxa are not shown. Colors of clades and locality numbers correspond to Fig. 1.

In general, our mtDNA-based tree topology of *Oligodon* (Fig. 2) corresponds well with the set of phylogenetic relationships obtained from previous studies (Green, Orlov & Murphy, 2010; Nguyen et al., 2020; Mirza, Bhardwaj & Patel, 2021; Das et al., 2022; Yushchenko et al., 2023a, 2023b). Both ML and BI topologies support the specimen of *O. taeniolatus* from Iran as a separate lineage sister to *Oligodon melaneus* Wall, 1909, *O. churahensis*, and Pakistani specimens *O. russelius* and *O*. cf. *churahensis*, with strong support from both analyses (0.97/92). Genetic p-distances in this clade varied from 7.8% (between *O. taeniolatus* and *O. melaneus*), 6.3% (between *O. taeniolatus* and *O. russelius*), and 5.7% (between *O. taeniolatus* and *O. churahensis*) based on cyt *b*. Together with *O. arnensis* they form the most basal clade (1/100), while the three samples of *O. taeniolatus* from India and Sri Lanka form a distinct clade together with *O. sublineatus* and *Oligodon calamarius* (Linnaeus, 1758) also with strong support (1.0/100). This topology renders *O. taeniolatus sensu* lato as paraphyletic. Notably, the sample of *O. taeniolatus* from Sri Lanka is divergent (2.2% p-distance based on 16S rRNA) from the Indian sample, which suggests additional undescribed diversity is present in *O. taeniolatus* outside of Middle and Southwest Asia. Distances of such a percentage based on 16S rRNA are more significant than the cyt *b* gene because 12S–16S rRNA evolves at a slower rate (Mueller, 2006). The newly collected Pakistan sample (CUHC 7904) identified by us as *O. russelius* is nested in a clade with two other Pakistan samples from GenBank identified as *O*. cf. *churahensis* by Mirza, Bhardwaj & Patel (2021) with high support (1.0/99). Low genetic divergence (*p* = 2.2%) exists between the three samples, suggesting they are conspecific. Additionally, the divergence between the Pakistani samples and the type series of *O. churahensis* from Himachal Pradesh, India is only 3.3% in cyt *b* gene (Table 4). The genetic distance between the sample re-identified as *O*. cf. *tillacki* and samples of *O. arnensis sensu* stricto was also low (*p* = 3.6%).

### Species distribution modeling

**Figure 3:**
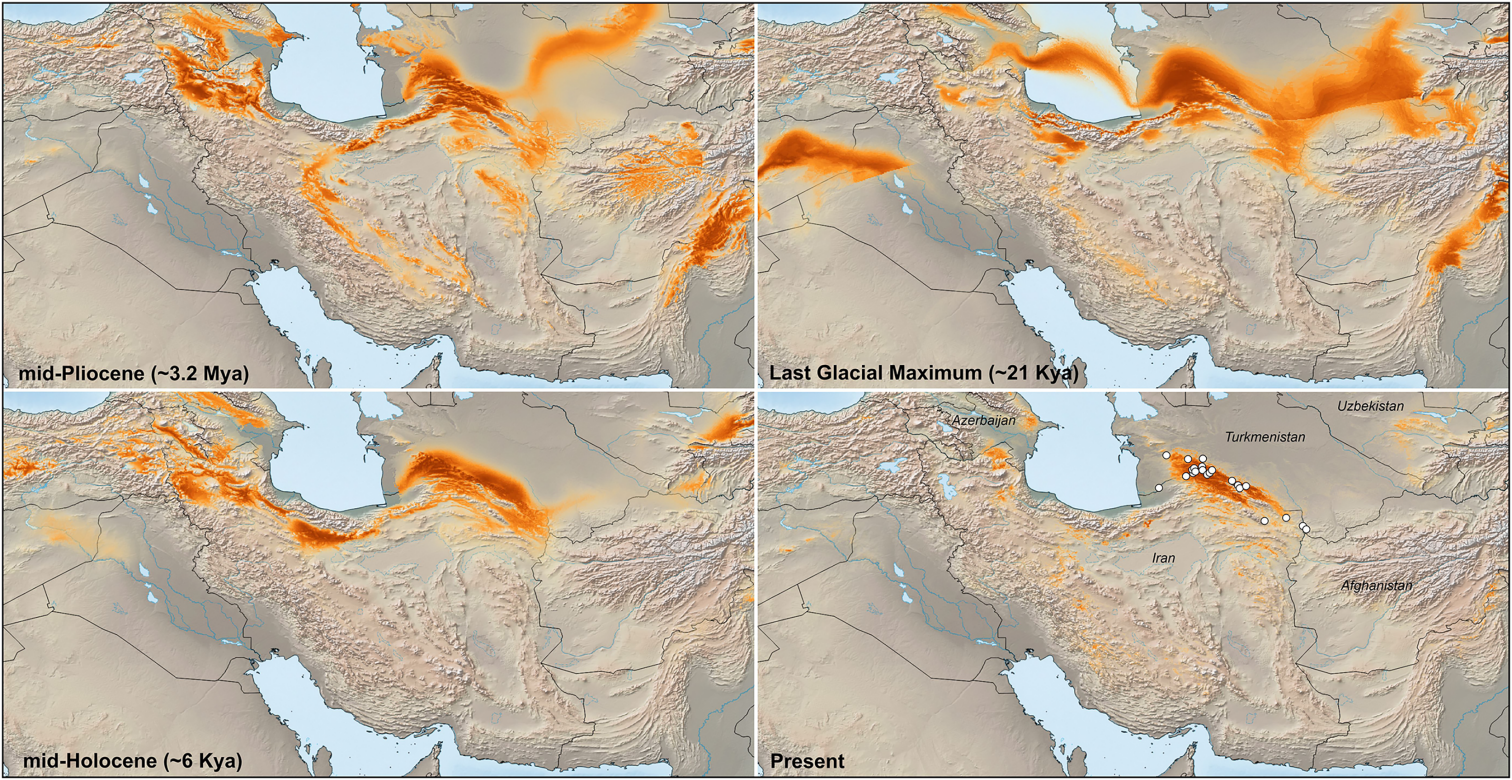
Species distribution model (SDM) map based on geolocation points of *Oligodon transcaspicus* comb. et stat. nov. from Iran and Turkmenistan. Darker red coloration indicates more suitable habitat, whereas lighter colors indicate less suitable habitat.

SDM maps based on geolocation points of *O. taeniolatus* from Iran and Turkmenistan are shown in Fig. 3. Variables that primarily account for species presence include landscape uniformity, mean temperature of the coldest quarter (Bio 11), slope, and precipitation seasonality (coefficient of variation) (Bio 15). The average performance of the MaxEnt SDMs for the replicate runs was estimated at AUC = 0.988. The predicted distribution of Middle and Southwest Asian *O. taeniolatus* experienced expansions and contractions across the Plio-Pleistocene and Holocene, indicating the species probably had a much wider range during historical times. The largest expansion of suitable habitat for the species mostly occurred during the last glacial maximum (LGM), with the distribution during this period including large portions of present-day Turkmenistan, Afghanistan, and Iran (near the southern portion of the Caspian Sea). Range reductions of *O. taeniolatus* started in the mid-Holocene, with the SDM maps predicting the most suitable habitat in the Köpet-Dag Mountain Range of northeast Iran and southwest Turkmenistan, and adjacent parts of Afghanistan, Azerbaijan, and western Iran (Fig. 3).

### Resurrection and revalidation of *Contia transcaspica* Nikolsky, 1902

Phylogenetically, the sample of *O. taeniolatus* from northern Iran is monophyletic and recovered in a separate clade within the broader *O. arnensis* species complex (defined here as *O. arnensis*, *O. churahensis*, *O. melaneus, O. russelius*, and *O. tillacki* per Bandara et al., 2022 and Das et al., 2022). Genetic distances between its closest relatives, *O. churahensis, O. melaneus*, and *O. russelius* range from 5.7%–7.8% (based on cyt *b*) and are typical of species-level divergences within *Oligodon* and other colubrid snakes. Based on morphology, all Iranian and Turkmen *O. taeniolatus* we examined are identical to the type specimen of *Contia transcaspica* (ZISP 9868), as well as past literature descriptions by Dotsenko (1984) and Latifi (1991). These specimens are distinct from Indian subcontinent *O. taeniolatus* in several features, the most obvious being the presence of only 5 (rarely 6) supralabial scales with only the 3^rd^ supralabial contacting the orbit, three (rarely 2) posterior temporal scales, narrow contact between the 4^th^ and 5^th^ supralabials, and the presence of brown transverse crossbars on the dorsal surface. Iranian and Turkmen *O. taeniolatus* are also distinct from members of the *O. arnensis* species complex based on several coloration and scalation features, especially the presence of 15 dorsal scale rows throughout the body (*vs*. 17–17–15). Since the combination of molecular and morphological evidence support the distinct status of Iranian and Turkmen *O*. “*taeniolatus*”, we resurrect the name *Contia transcaspica* from the subjective junior synonymy of this species. This decision maintains the monophyly of *O. taeniolatus sensu* stricto and restricts its range to the Indian Subcontinent. The Iranian and Turkmen populations from the Köpet–Dag Mountain Range should now be referred to as *Oligodon transcaspicus* **comb. et. stat. nov.** and a detailed redescription, including the holotype of *Contia transcaspica*, is provided below.

**Figure 4:**
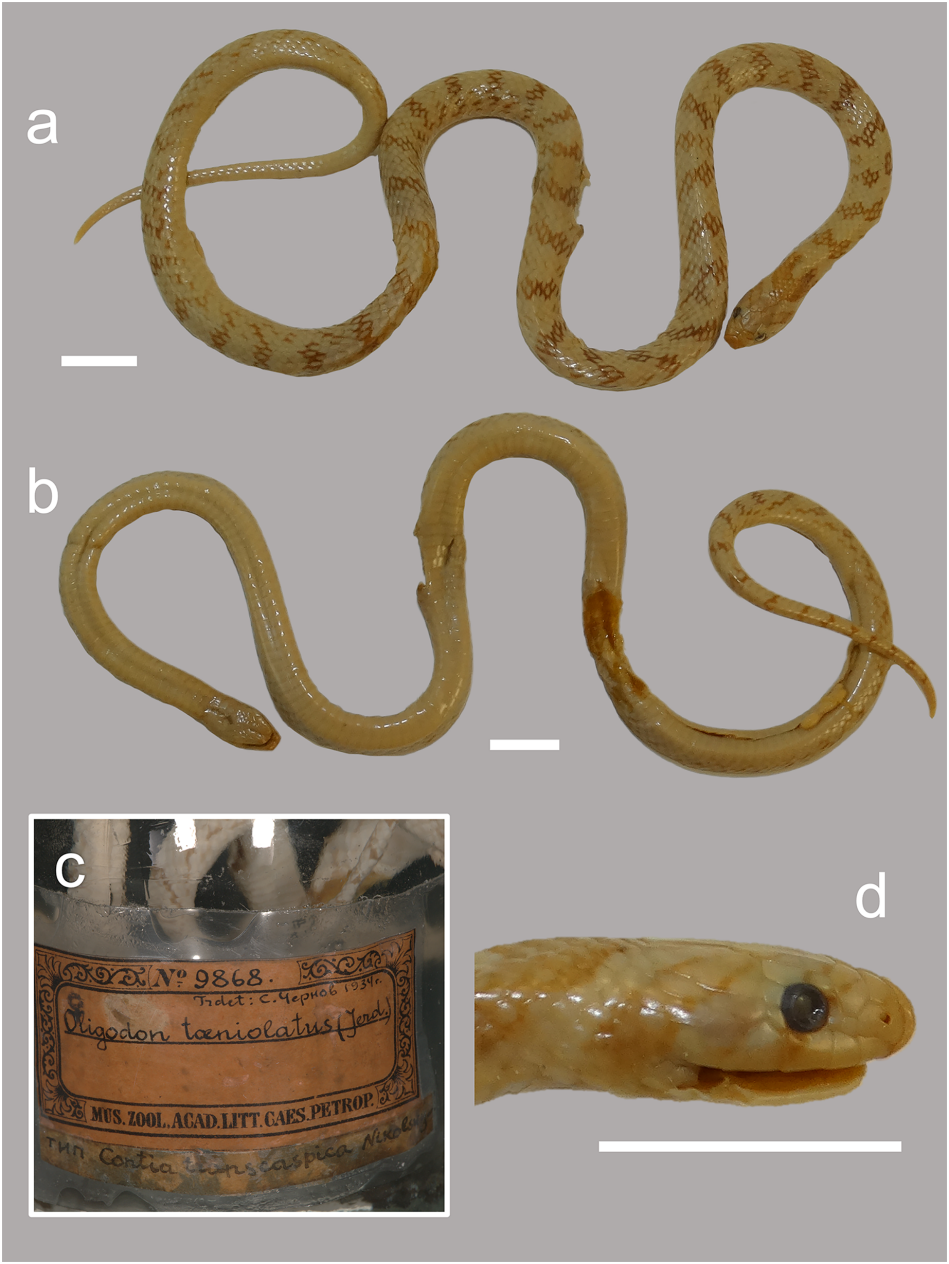
(A–D) Photographs of the preserved holotype specimen of *Contia transcaspica* (ZISP 9868) now *Oligodon transcaspicus* comb. et stat. nov. from “Köpet–Dag, Transcaspia”. Scale bars equal 10 mm. Photos by Konstantin D. Milto.

**Figure 5:**
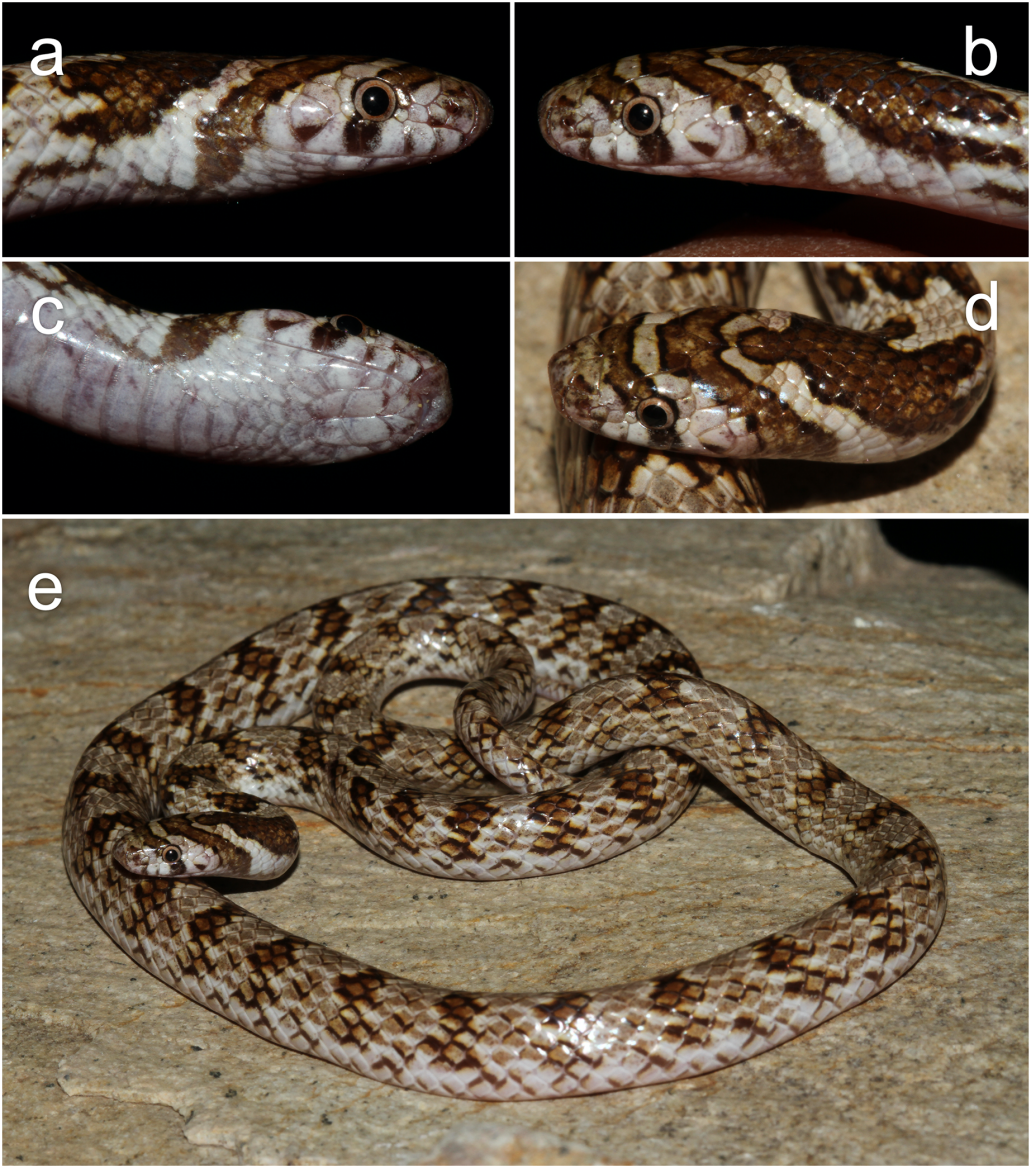
Live photographs of *Oligodon transcaspicus* comb. et stat. nov. (ZMMU Re-16687; field number RAN-3264) from Khorasan Province, Iran. (A) Right lateral, (B) left lateral, (C) ventral, (D) ventral views of the head, and (E) general habitus. Photographs by Roman A. Nazarov.

**Figure 6:**
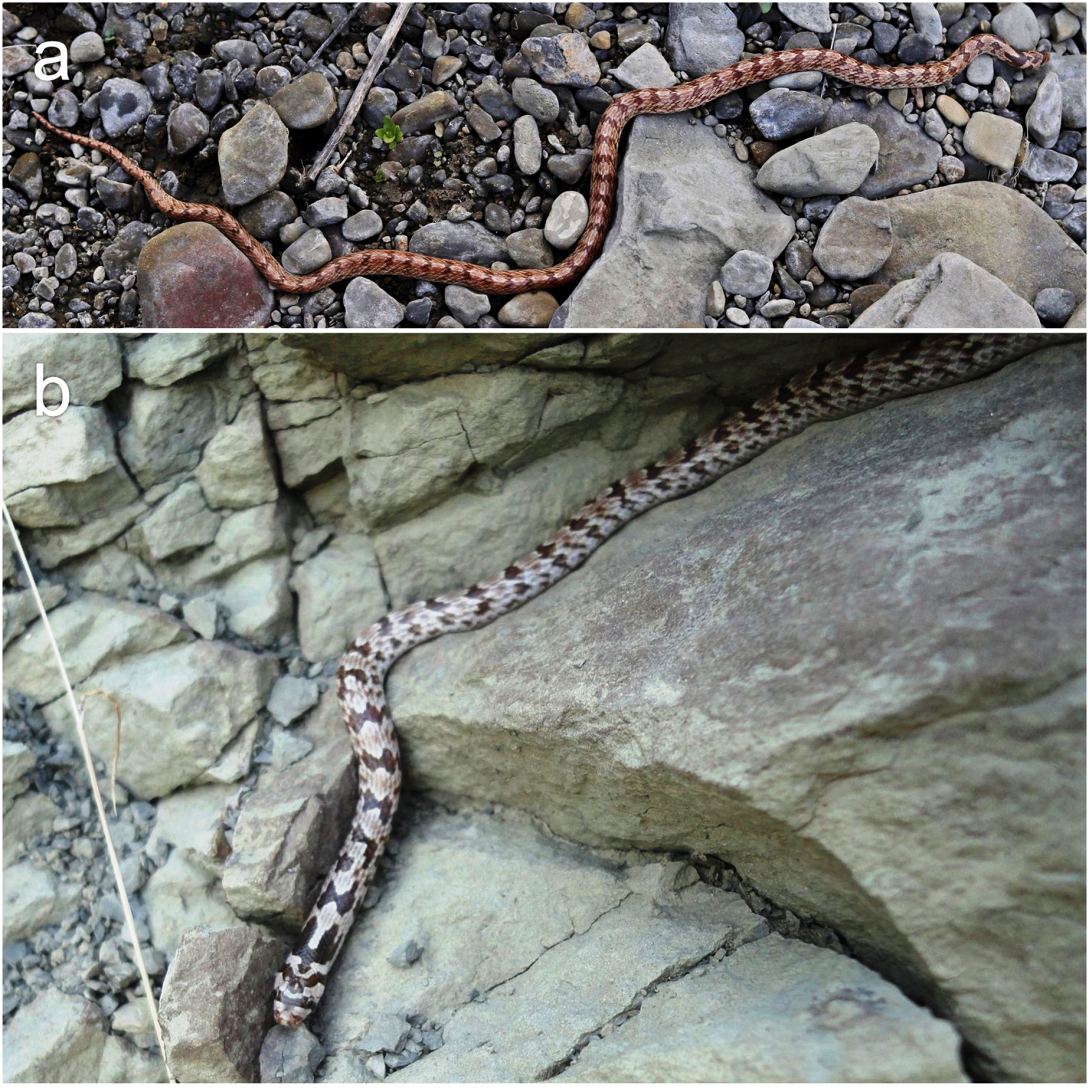
Two unvouchered specimens of *Oligodon transcaspicus* comb. et stat. nov. Specimens photographed from (A) Podere, Sumbar Valley, Turkmenistan and (B) Seqiz-Khan Gorge, Turkmenistan. Photographs taken by Alexander V. Pavlenko.

***Oligodon transcaspicus* (Nikolsky, 1902)** **comb. et stat. nov.**

(Figures 4–6; Table 2)

*Contia transcaspica*: Nikolsky (1902: 839–840). *Holotype*: ZISP 9868 collected from “Kopet-Dag, Transcaspia” (now Köpet–Dag Mountain Range, near Ashgabat, Ahal Province, Turkmenistan) by N. Kinschin in 1901, Nikolsky (1903a: 11–13), Bobrinskoy (1923: 8), Chernov (1935: 351) (in part), Welch (1983: 77) (in part), Green (2010: 139) (in part), Bandara et al. (2022: 68) (in part), Uetz et al. (2023) (in part).

*Oligodon taeniolatus* (in part): Chernov (1935: 351), Filippov (1947), Terentjev & Chernov (1949), Bogdanov (1962), Brück (1968: 201), Nurgeldyev, Shammakov & Atayev (1970: 187–190), Rustamov & Atayev (1976: 47–53), Bannikov et al. (1977), Atayev, Gorelov & Shammakov (1978), Szczerbak (1979: 68–70), Welch (1983: 77), Dotsenko (1984: 23–26), Atayev (1985), Szczerbak, Khomustenko & Golubev (1986: 68–70), Latifi (1991: pl. 52 & 117), Rustamov & Sopyev (1994: 224), Atayev, Rustamov & Shammakov (1994: 337), Szczerbak (1994: 312), Latifi (2000), Ananjeva et al. (2006: 175), Green (2010: 139), Rustamow (2011), Safaei-Mahroo et al. (2015: 280), Shestopal & Rustamov (2018a: 40), Orlov et al. (2018: 58–67), Rajabizadeh (2018: 242).

**Holotype**. ZISP 9868, adult female from “Köpet–Dag, Transcaspia” (=now Köpet–Dag Mountain Range, near Ashgabat, Ahal Province, Turkmenistan) collected by N. Kinschin in 1901 (Fig. 4).

**Referred specimens**. **Turkmenistan:** Ahal Province. CAS 180042, adult female from “the Iran border south of Goalon, Ashkabad (Ashkhabad) Region” collected in May 1989 by Soviet Border Patrol (obtained by J. R. Macey); ZMMU Re-5589, subadult female from “Karanki”, collected on 1 May 1979; ZMMU Re-6155, juvenile male from “Aidere” unknown collection date; ZMMU Re-7318, adult female from “Chuli, Geok-Tepe” collected on 20 June 1990; ZISP 18334, juvenile of undetermined sex from “Geok-Tepe”, collected on May 1971; ZISP 18976, subadult male from “Geok-Tepe”, collected on 05 August 1968. **Iran:** Khorasan Province. ZMMU Re-16687 (field number RAN-3264), adult male from Bazangan Lake, Razavi (36.3044°N, 60.4751°E, WGS 84; 900–950 m a.s.l.) collected by Roman A. Nazarov and Mahdi Rajabizadeh on 27 May 2017. See Table S1 for more details.

**Diagnosis**. A kukri snake in the genus *Oligodon* that is distinguished from all other congeners by the following combination of morphological characters: (1) 7–9 maxillary teeth, with the posterior two teeth enlarged and blade-like; (2) dorsal scales in 15–15–15 rows; (3) cloacal plate divided; (4) ventral scales 179–188 in males, 193–214 in females; (5) subcaudals 48–52 in males, 44–51 in females; (6) total body scales 232–240 in males, 240–266 in females (232–266 scales combined); (7) subcaudal ratio 0.203–0.224 in males, 0.177–192 in females; (8) almost always five supralabials (sometimes six according to Latifi, 1991), with only the 3^rd^ supralabial contacting the orbit; (9) loreal and preocular scales present, presubocular sometimes present; (10) posterior temporal scales three (rarely 2), with the lowest temporal causing 4^th^ and 5^th^ supralabial to contact narrowly; (11) dorsal color pattern beige or light brown with of 42–57 dark transverse crossbars on body and 12–17 crossbars on the tail; (12) dorsal color pattern on tail resembling the rest of the body, with no vertebral stripe.

**Comparisons**. We compare *Oligodon transcaspicus* **comb. et stat. nov.** to all species of *Oligodon* found in Middle and Southwest Asia, which may be confused with this species, particularly *O. taeniolatus sensu* stricto, with which it was previously confused with, and members of its sister clade in the *O. arnensis* species complex. We base our comparisons primarily on data from the following literature sources (Wall, 1921, 1923; Smith, 1943; Khan, 2002; Mirza, Bhardwaj & Patel, 2021; Bandara et al., 2022) as well as data from our own examined specimens (Material S1). *Oligodon transcaspicus* **comb. et stat. nov.** can be morphologically assigned to the genus *Oligodon* by having a subcylindrical body, enlarged blade-like maxillary teeth, two prefrontals and internasals present, a blunt and subterminal shaped rostral scale, length of rostral scale visible from above two times as long as the internasal suture, and two temporal scales bordering the edge of the parietals (generic diagnosis modified from Wall, 1923).

The closest relatives of *O. transcaspicus* **comb. et stat. nov.** include members of the *O. arnensis* species complex. *O. transcaspicus* **comb. et stat. nov.** can be distinguished from *O. arnensis sensu* stricto by having 15 dorsal scale rows (*vs*. 17–17–15), 179–202 ventrals in both sexes (*vs*. 164–188 in both sexes), loreal scale present (*vs*. absent), 5–6 supralabials (*vs*. 7–8, rarely 6), usually three posterior temporal scales (*vs*. always 2), 7–9 maxillary teeth (*vs*. 12–16), and irregular dorsal mottling with 42–57 transverse body crossbars (*vs*. dorsum immaculate, less than 20 black body bands). *O. transcaspicus* **comb. et stat. nov.** can be distinguished from *O. churahensis* (later synonymized with *O. russelius*) by having 15 dorsal scale rows (*vs*. 17–17–15), 179–202 ventrals in both sexes (*vs*. 170–175 in both sexes), 5–6 supralabials (*vs*. 7–8), usually three posterior temporal scales (*vs*. always 2), and irregular dorsal mottling with 42–57 transverse body crossbars 1.5–3.0 dorsal scales wide and 12–16 tail crossbars (*vs*. 37–45 broad black body crossbars, all edged with a cream color, 1.0–2.0 dorsal scales wide and 9–11 tail bars). *O. transcaspicus* **comb. et stat. nov.** is distinguished from *O. melaneus* by having 15 dorsal scale rows (*vs*. 17–15–15), 5–6 supralabials (*vs*. usually 7, rarely 6), usually three posterior temporal scales (*vs*. always 2), and irregular dorsal mottling with 42–57 transverse body crossbars 1.5–3.0 dorsal scales wide and 12–16 tail crossbars (*vs*. black dorsum without conspicuous blotches or markings and a distinct blue ventral coloration in life). *O. transcaspicus* **comb. et stat. nov.** can be distinguished from *O. russelius* by having 15 dorsal scale rows (*vs*. 17–17–15), 5–6 supralabials (*vs*. 7–8, rarely 6), usually three posterior temporal scales (*vs*. always 2), and irregular dorsal mottling with 42–57 transverse body crossbars 1.5–3.0 dorsal scales wide (*vs*. dorsum immaculate with narrow black body bands or crossbars usually edged with white, 1.0–2.0 dorsal scales wide). *O. transcaspicus* **comb. et stat. nov.** can be distinguished from *O. tillacki* by having 15 dorsal scale rows (*vs*. 17–17–15), 5–6 supralabials (*vs*. 7–8), usually three posterior temporal scales (*vs*. always 2), and irregular dorsal mottling with 42–57 transverse body crossbars 1.5–3.0 dorsal scales wide (*vs*. dorsum immaculate with 25–35 broad black body bands 4.0–6.0 dorsal scales wide).

In addition, *O. transcaspicus* **comb. et stat. nov.** can be distinguished from *O. taeniolatus*, which it was previously confused with, by having 5 (rarely 6) supralabials (*vs*. 7–8, rarely 6) with only the 3^rd^ supralabial contacting the orbit (*vs*. usually the 3^rd^ and 4^th^ in contact with orbit), two posterior supralabials (4^th^ and 5^th^) in narrow contact due to the lowest posterior temporal scale abutting the two scales (*vs*. all supralabials in broad contact), temporal scale formula usually 1 + 3 (*vs*. temporals 1 + 2), and a dorsal color pattern with irregular transverse crossbars and no distinct vertebral stripe on the body or tail (*vs*. dorsal color pattern variable, but when crossbars or body blotches are present they are always irregular and non-transverse in shape, and a thin vertebral stripe on the body and tail is usually present).

**Redescription of the holotype (ZISP 9868; Fig. 4)**. Adult female specimen in good condition after 121 years of preservation. Small portion of midbody in fair condition, large ventral incision on the posterior third of the body, ending at the vent (Fig. 4). SVL 304 mm, TailL 53 mm (TotalL 357 mm). HeadL 9.6 mm, HeadW 5.6 mm, SnL 2.6 mm, EyeL 1.4 mm, FrontalL 3.2 mm. TailLR 0.148, HeadW/HeadL 0.58, SnL/HeadL 0.29, EyeL/SnL 0.54, EyeL/HeadL 0.15. Body elongated and cylindrical; head ovoid, slightly distinct from neck; snout narrowing in dorsal view, slightly depressed towards the tip, rostral round and slightly protruding in lateral profile; snout tip subterminal near mouth; eyes moderately sized compared to head, pupil round; nostrils small and subelliptical, pointed in lateral view; mouth flat with lips curled upwards posteriorly relative to the last supralabial; tail gradually tapering to a sharp terminal scute.

Rostral distinctly enlarged, wider than high, triangular in dorsal view, partially separating anterior half of internasals; posterior scale suture of rostral bordering internasals “deep-V” shaped, posterior vertex of rostral in-line with nostrils narrowly obtuse angled (~99°); internasals subpentagonal, wider than long, internasal suture equal in length compared to prefrontal suture; prefrontals subhexagonal, wider than long, wider and longer than internasals; frontal subpentagonal, shield shaped, anterior suture with prefrontals concave; frontal longer than prefrontals; eyes placed posterior relative to the anterior edge of the frontal; angle formed by posterior sutures producing the vertex of the frontal a right angle, almost acute (~90); supraoculars subrectangular, longer than wide, shorter in length than frontal; parietals subpentagonal, longer than wide, wider than parietal suture, posterior sutures bordering occipital region strongly concave; length of parietals roughly equal to length of frontal; parietal suture shorter than frontal; parietal suture shorter than length of frontal; anterior parietal angle formed by the sutures between the parietal/frontal and the suture between the supraocular/parietal a broad obtuse angle (~130°) with its lateral ray pointing posterolaterally. Nasal scale rectangular, longer than wide, fully divided; loreal 1/1, small and square shaped, slightly wider than than long, less than half the size of the nasal; supralabials 5/5; 3^rd^ supralabial in contact with orbit; 5^th^ supralabial largest, first supralabial smallest; all supralabials in broad contact, except for supralabials 4–5, which are in narrow contact due to abutting posterior temporal; preoculars 1/1, wider than long; presubocular 1/1, smaller than preocular; postoculars 2/2, uppermost postocular largest; anterior temporal 1/1; posterior temporals 3/3, bottommost temporal large and subpentagonal in shape, blocking the 4^th^ and 5^th^ supralabial from broadly contacting each other; infralabials 7/7 in all specimens, first in contact with each other, 4/4 infralabials in contact with anterior chin shields; mental subtriangular, wider than long; small mental groove present, starting from border of 1^st^ infralabial and mental terminating at the posterior chin shields; length and width of both chin shields equal in size.

Dorsal scale rows 15–15–15, smooth throughout without apical pits; ventral scales 202; subcaudals 47, paired; total body scales 250; subcaudal ratio 0.19; cloacal plate divided; tail tip a sharp pointed scute. Maxillary teeth 7, posterior two teeth enlarged and blade-like.

**Coloration in preservation**. After 121 years in preservation fluid, dorsal ground color cream, margins of dorsal scales brown forming indistinct and irregular mottling most heavily concentrated along the flanks; 47 indistinct transverse crossbars across the dorsum, irregular, broadest vertebrally, narrowing laterally, around 1.5–2.0 dorsal scales at their widest; along tail 12 more transverse crossbars of similar color. Dorsal portion of head cream with an indistinct beige ocular-bar edged with brown starting along 3^rd^ and 4^th^ supralabial scale, crossing through each eye and covering the first half of the supraocular, prefrontals and anterior frontal suture; a second beige and brown-edged temporal bar originating near the gulars and first dorsal scales, extending through the posterior portion of the fifth supralabial before meeting medially at the parietals and frontal forming a “V” shaped marking; brown nuchal chevron present starting at the parietal suture as a small lanceolate tip, broadening along the nape as a thick tripartite shaped blotch, extending laterally on each side of the flanks before ending near the edge of the ventral scales. Ventral surface immaculate cream, faint beige spotting and mottling along the edge of each ventral scale, more prominent along the tail.

**General description and variation (Figs. 5, 6)**. The additional six specimens examined agree with the description of the holotype in most aspects of coloration, scalation and morphometric characteristics. SVL 145–312 mm in male, 267–361 mm in females; TailL 27–70 mm in male, 42–64 mm in females; TotalL in male 195–382 mm, 309–425 mm in females. The largest specimen is an adult female (ZMMU Re-7318) with a SVL of 361 mm and TailL of 64 mm. HeadL 5.9–9.6 mm; HeadW 3.6–6.0 mm, SnL 2.3–3.5 mm, EyeL 0.8–1.4 mm, FrontalL 1.4–3.2 mm. TailLR 0.157–0.183 in males, 0.136–0.159 in females, HeadW/HeadL 0.53–0.72, SnL/HeadL 0.27–0.42, EyeL/SnL 0.34–0.54, EyeL/HeadL 0.12–0.15. Body elongated and cylindrical, slightly robust along midbody in some specimens; head ovoid, slightly distinct from neck; snout narrowing in dorsal view, slightly depressed in lateral view; head oblong in lateral profile; snout tip subterminal near mouth; eyes moderately sized when compared to the head; nostrils pointed in lateral view; mouth flat, lips slightly curled posteriorly relative to the last supralabial; tail tapering to a sharp terminal scute.

Rostral distinctly enlarged, wider than high, triangular in dorsal view, extending posteriorly and separating the first half of the internasals from contacting; posterior suture of rostral bordering the internasals “deep-V” shaped, posterior vertex of rostral in-line with nostrils obtuse angled (95°–115°); internasals subpentagonal, wider than long, internasal suture slightly longer or equal to prefrontal suture; prefrontals subhexagonal, wider than long, larger than internasals; frontal subhexagonal, shield shaped, anterior suture usually concave; the posterior suture of the frontal of one specimen (ZMMU Re-16687) has a pair of pores near the border of the parietals and at the center of the scale; frontal roughly twice the length of prefrontals; supraoculars subrectangular, longer than wide, shorter in length and width than frontal; eyes placed posterior relative to the anterior edge of the frontal; angle formed by sutures producing the posterior vertex of the frontal right or acute-angled (80°–90°), making the posterior portion of the frontal appear acuminate in dorsal view; parietals subpentagonal, posterior portion concave, occasionally squared; parietals slightly longer than wide, wider than parietal suture; length of parietals equal or slightly longer than length of frontal; parietal suture shorter than frontal length; anterior parietal angle formed by the sutures between the parietal/frontal and the suture between the supraocular/parietal a broadly obtuse (120°–130°), with the lateral ray pointing posterolaterally. Nasal scale rectangular to square shaped, fully divided, longer than wide; loreal 1/1, square, around one fourth or half the size of the nasal; supralabials 5/5 in all examined specimens, although Latifi (1991) mentions specimens with 6 supralabials; 3^rd^ supralabial always in contact with orbit; 5^th^ supralabial largest, 1^st^ supralabial smallest; all supralabials in broad contact with each other except for supralabials 4–5, which contact narrowly due to abutting posterior temporal; preoculars 1/1, uppermost preocular larger; presubocular 1/1 present in four specimens (including holotype), absent in other three specimens; postoculars 2/2, uppermost postocular largest; anterior temporal 1/1; posterior temporals 3/3 (in one specimen, CAS 180042, 3/2); bottommost temporal largest, subpentagonal, blocking the 4^th^ and 5^th^ supralabial from broad contact; infralabials 7/7 in all specimens, the first pair in contact with each other, 4/4 infralabials in contact with anterior chin shields; mental subtriangular, wider than long; a small mental groove present, starting at the border of the 1^st^ infralabial and terminating at the posterior chin shields; length and width of both chin shields equal in size.

Dorsal scale rows 15–15–15, smooth throughout and without apical pits; ventral scales 179–188 in males, 193–202 in females; subcaudals 48–52 in male, 44–51 in females; total body scales 232–240 in male, 240–266 in females; subcaudal ratio 0.203–0.224 in males, 0.177–192 in females; anal plate divided; tail tip tapers to a sharp tip. Maxillary teeth 7 in two specimens (including the holotype) and 9 in one specimen (CAS 180042), posterior two teeth enlarged and blade-like. Teeth unavailable for examination in other specimens. We did not examine the hemipenis of any specimens as all organs were retracted. The structure of hemipenes is a rather conservative feature. Therefore, despite the lack of any information about hemipenial morphology of *O. transcaspicus* **comb. et stat. nov.**, we expect that the fully everted and expanded hemipenes of this species will likely share the same structure with its closest relatives in the *O. arnensis* species complex, namely a short and slightly bilobed hemipenes with spinous calyces and a simple sulcus spermaticus. Our sample size is too low to make statistical comparisons between sexes, however a few instances of putative sexual dimorphism are noted. First, the number of ventral scales appear to be higher in the single male specimen. The male specimens also have a higher number of subcaudals, TailLR ratio and subcaudal ratio than the females. The number of total body scales appears to be higher in females.

The dorsum of all specimens in preservative is cream or beige with well-defined brown mottling concentrated along the flanks; white mottling is also present on the vertebral region in some specimens; 42–57 light brown transverse crossbars on the body, each bar weakly edged with white; all crossbars more well-defined compared to the holotype, irregular, 1.5–3.0 vertebral dorsal scales in length, broadest vertebrally and narrowest laterally; margins of the dorsal scales within each crossbar dark brown or black; 12–16 crossbars present on the tail, similar in coloration. No sexual dimorphism was noted in the number of body or tail crossbars. Dorsal surface of the head tan to cream, anteroventral portions of the snout gray-brown in some specimens; gray-brown mottling occasionally present along the supralabials and underside of head; the position of the ocular and temporal bars are consistent amongst all specimens, but vary in coloration from brown to dark-brown and are usually edged with black; the nuchal chevron in some specimens is lanceolate-shaped at its anterior origin along the parietals, but in two specimens it is blunt or obtuse, terminating before the ventral surface along the nape as a thick triangular shaped blotch. No vertebral stripe is present along the tail in preserved specimens. The ventral surface is tan to cream and usually immaculate, however in three specimens the dorsal crossbars extend onto the lateral sides of the ventral scales. The color in life based on ZMMU Re-16687 (Fig. 5) and two specimens photographed in Turkmenistan by A. V. Pavlenko (Fig. 6) resembles the coloration in preservative, but the dorsal patterning and blotching are more pronounced. In these specimens, the dorsum is brown to reddish-brown with white mottling concentrated on the vertebral region and dark-brown mottling concentrated on the flanks; the transverse bars are brown and have small dark-brown edges on the dorsal scales of each bar; the nuchal chevron and ocular bars are darker than the crossbars on the dorsum; the eyes have a gold-brown iris, pupils black; ventral surface and areas between head markings plain white.

**Distribution and natural history**. The known distribution of *Oligodon transcaspicus* **comb. et stat. nov.** is summarized in Fig. 1 and Table S1. This species is currently known from the Köpet–Dag region of northern Iran and southern Turkmenistan. In Turkmenistan, it is known from the present-day Balkan, Ahal and Mary provinces. The distribution of the species in Turkmenistan was recently reviewed by Orlov et al. (2018). They report *O. transcaspicus* **comb. et stat. nov.** from ten localities in the foothills of the Köpet–Dag Mountains: Balkan and Ahal Provinces (Danata spring; Eldere Gorge north from Kara–Kala; Chandyr Valley; Makhmumkala village; Kara–Elchi and Eishem gorges; Aidere, Kurygol; Arvaz Valley; 7 km northwards from Saivan; Firuza and Chuli villages; Shamly, near Babazo) and from two localities in Mary Province (Dana–Germab spring, and Nardyvanly spring; environs of Badkhyz). In Iran, *Oligodon transcaspicus* **comb. et stat. nov.** is known definitively from Razavi Khorasan Province from two localities (ZMMU Re-16687 from Bazangan Lake; and another sight record we confirm from ~5 km SW of Mashahd), and Golestan Province (town of Dashliburun (Dashil Borun)). The Golestan Province records originates from Latifi (1991), who reported the locality as “Mazandaran Province (Ghonbad Kavoos) … in Dashley Boron region”. At the time of Latifi’s writings, the locality “Ghonbad Kavoos… Dashley Boron” (the romanized spelling of Gonbad-e Kavus county) was included within Mazandaran Province before being separated into Golestan Province in 1997. We here confirm this locality in Golestan Province as “Dashliburun” (sometimes spelled as “Dashil Boron”), close to the Turkmenistan border. Rajabizadeh (2018) reported the species in Northeastern Iran close to border of Turkmenistan from eastern Golestan Province to north of Khorasan Razavi Province. Latifi (2000) and Safaei-Mahroo et al. (2015) also reported this species from North Khorasan, and from Zanjan and West Azarbaijan Provinces, although the occurrences in western Iran require further verification (see Discussion).

Several authors have provided insights on the natural history of *O*. *transcaspicus* **comb. et stat. nov.** (Dotsenko, 1984; Atayev, 1985; Szczerbak, Khomustenko & Golubev, 1986; Rustamov & Sopyev, 1994; Szczerbak, 1994; Atayev, Rustamov & Shammakov, 1994; Orlov et al., 2018). Two individuals photographed by A. Pavlenko were found in the daytime amongst rocky outcrops (Fig. 6); however, Orlov et al. (2018) noted specimens were predominately nocturnal. These authors report that *O*. *transcaspicus* **comb. et stat. nov.** was most abundant between elevations of 400–700 m and note a few specimens which were found at higher altitudes up to 1,000 m. Most of the specimens recorded were found on the surface or under rocks at the base of gradually sloping mountain gorges, mudstone channels, riverbeds and open habitats with shrubs and tree vegetation that maintain enough moisture. Based on this, Orlov et al. (2018) posited that the activity period of *O*. *transcaspicus* **comb. et stat. nov.** is highly dependent on recent precipitation and relative humidity. Ananjeva et al. (2006) noted that *O*. *transcaspicus* **comb. et stat. nov.** was a rare species in Turkmenistan, although the summary of observations by Orlov et al. (2018) may indicate it is more common than currently ascertained and may be difficult to observe in the field unless specific seasonal and weather conditions are met. Like other *Oligodon*, this species probably feeds on reptile and bird eggs, and may also consume small lizards, frogs, and small mammals. Atayev, Rustamov & Shammakov (1994) and sources therein record this species laying one to two eggs between the months of May and June, suggesting the reproductive season is in the spring and early summer.

**Remarks**. Most sources that mention *O. transcaspicus* **comb. et stat. nov.** have cited Nikolsky (1903a) as the source of original description. However, we have discovered an earlier book that was also authored by Nikolsky describing *Contia transcaspica* dated to 1902. Thus, the year of original description and its publication source should be fixed, and we do so here in the present article.

**Etymology**. The species epithet “*transcaspicus*” is a latinized toponymic adjective in genitive singular and given in reference to the type locality of this species, which during the time of its description, was called the Transcaspian Region (Zakaspiyskaya Oblast or Zakaspiyskiy Krai) and was part of the Turkestan Governor–Generalship of the Russian Empire. The Transcaspian Region later became known as Turkmenistan during its time as a constituent republic of the Soviet Union (Turkmen S.S.R.) and now as an independent nation. The epithet is fixed to agree with the gender of the generic name *Oligodon*, which is masculine. We recommend the English common name “Köpet–Dag kukri snake” for this species, followed by the Russian, Farsi, and Turkmen common names “Zakaspiyskiy oligodon”, “داغ کپه مار لوس ” (*Loos Mār-e Kopet Dagh*), and “Goňurja ýylanjyk”, respectively (see Rustamow, 2011; Rajabizadeh, 2018).

**Figure 7:**
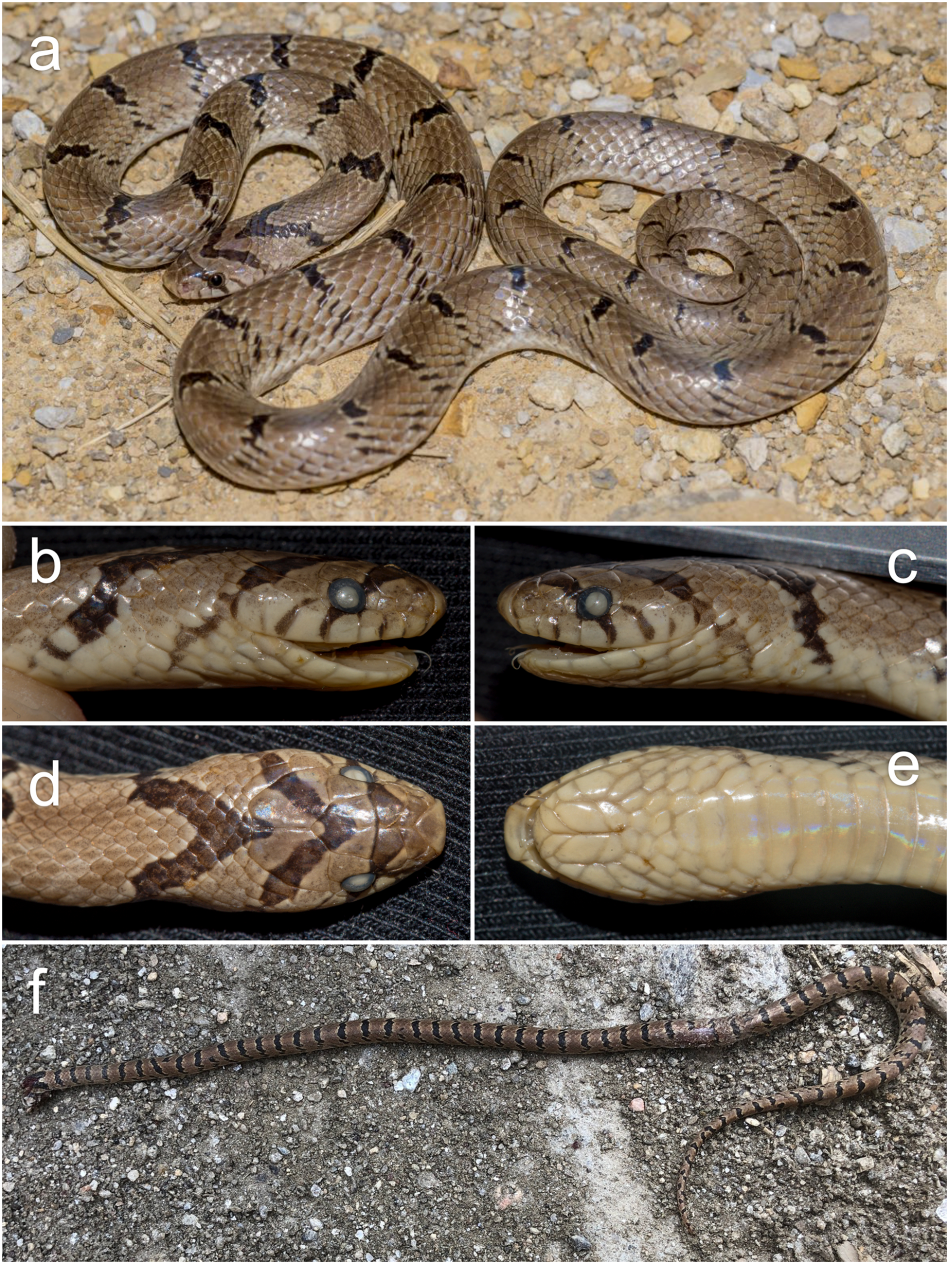
Photographs of *Oligodon russelius* from Pakistan (A–E) and Afghanistan (F). Shown in (A)–(E) is specimen CUHC 7904 from Kallar Kahar, Punjab Province, Pakistan, showing (A) general habitus in life, (B) right lateral, (C) left lateral, (D) dorsal, and (E) ventral views of the head in preservative. Shown in (F) is a cropped image of *O. russelius* from Kunar Province, Afghanistan observed on iNaturalist (obs. 110932106, user mohammadfarooq). Photographs (A–E) by Daniel Jablonski, photograph (F) taken by Mohammad Farooq from iNaturalist.org.

**The taxonomic status of *Oligodon “arnensis”* in Pakistan**. Because the revision of *O. arnensis sensu* auctorum by Bandara et al. (2022) only focused on material from India and Sri Lanka, we use this section to review the status of populations in neighboring Pakistan. We examined one specimen of *O*. “*arnensis*” (CUHC 7904) recently collected by DJ and RM on 16 September 2018 from Kallar Kahar, Punjab Province, Pakistan (32.7695°N, 72.7065°E, 613 m a.s.l.). Based on the presented mtDNA phylogeny, this specimen was recovered in a clade with two additional samples from Pakistan that were previously identified as *O*. cf. *churahensis* by Mirza, Bhardwaj & Patel (2021). A brief description of CUHC 7904 is provided here (Fig. 7): adult male, SVL 345 mm, TailL 65 mm (TotalL 410 mm; TailLR 0.159) 16–17–15 dorsal scale rows, 183 ventrals, 49 subcaudals (233 total body scales), subcaudal ratio of 0.21, 1/1 loreal present, 1/1 divided nasal, 7/7 supralabials with the third and fourth in contact with the eye, 7/7 infralabials, and an immaculate brown dorsum with 37 black crossbars across the body and 13 tail bars approximately 1.0–1.5 dorsal scales wide at midbody and interspaces between each bar approximately 4.0 dorsal scales long. In addition to this specimen, past authors have also recorded *O. “arnensis”* specimens from Pakistan (Minton, 1962; Minton, 1966; Mertens, 1969; Khan, 2002). Both Minton (1962) and Khan (2002) figure an *O. “arnensis”* (with Khan, 2002 swapping the images of *O. “arnensis”* and *O. taeniolatus* by mistake) bearing small narrow black crossbars with white edges. Based on these characteristics, along with other scalation features (see Table 3), the literature descriptions of Pakistani *O. “arnensis”* are very similar to CUHC 7904 and specimens *O. russelius sensu* Bandara et al. (2022). Therefore, based on our specimen and previous reports of *O. “arnensis”*, we refer the northern Pakistan populations of *O. “arnensis”* to *O. russelius*.

**Records of *Oligodon* in Afghanistan**. Literature reports of *O. “arnensis”* and *O. taeniolatus* from Afghanistan are sparse but have been the subject of confusion for decades. Brück (1968) first reported a juvenile kukri snake he identified as *O. taeniolatus* from “dem Gebiete um Djelalabad” (=vicinity of Jalalabad). This record was plotted by Sindaco, Venchi & Grieco (2013) but rejected due to imprecision by Wagner et al. (2016b). Although Brück (1968) identified this specimen as *O. taeniolatus*, data he provided for this specimen indicates it bears 17 dorsal scale rows, contra the 15 dorsal scales normally observed in *O. taeniolatus*. This was noticed quickly by Král (1969), who subsequently corrected its identification to *O. arnensis*. Nevertheless, both Sindaco, Venchi & Grieco (2013) and Wagner et al. (2016b) record *O. arnensis* and *O. taeniolatus* as inhabitants of Afghanistan, unaware that the records refer to the same specimen (MMB 28497). Brück (1968)’s account provided additional morphological data that allow us to confirm its identification. He notes that the specimen bears the following features: juvenile (unsexed, but based on relative tail length and subcaudal ratio, possibly an immature female), SVL 153 mm, TailL 24 mm (TotalL 177 mm, TailLR 0.136), dorsal scales in 17 rows, 198 ventrals, 44 subcaudals, (243 total body scales, subcaudal ratio 0.181), 7/7 supralabials (3–5 contacting eye), 6/6 infralabials, one preocular, two postoculars, 1 + 2 temporals, and 49 dark crossbars across the body and tail. These characteristics confirm that the specimen should not be identified as *O. taeniolatus* and that Král’s re-identification as *O. “arnensis”* was correct (see Table 3 for more comparisons). A second *Oligodon* record from Afghanistan was recently documented on the citizen science platform iNaturalist (2022). This specimen (obs. 110932106, user mohammadfarooq) was observed from Dara–i–Pech district, Kunar Province, Afghanistan (35.0553°N, 70.9561°E; 1,700–1,800 m a.s.l.) on 9 April 2022. While photographs of the specimen are too poor in quality to discern any scalation features, its color pattern consisting of approximately 51 black crossbars and 13 tail bars (64 total crossbars) greatly resembles *O. “arnensis”*, specifically *O. russelius* (and to some extent *O. churahensis*, but see Discussion). In conjunction with our re-identification of Pakistani populations, we conclude that the Afghanistan populations previously recorded as *O. arnensis* (Brück, 1968; Král, 1969; Khan, 2002; Wagner et al., 2016b) should also represent *O. russelius*. All records of this species from the country are restricted to the western foothills of the Hindu Kush.

The only other report of an Afghan *Oligodon* is a record of *O. taeniolatus* noted by Wagner et al. (2016b) from “Kars, Kandahar Province” based on specimen USNM 194971. We attempted to trace the voucher of this specimen and discovered that the museum catalog number USNM 194971 actually refers to a scincid lizard *Eurylepis taeniolatus* Blyth, 1854a, and not a kukri snake. No collection records of *Oligodon* from Afghanistan have been found within the USNM collections ledger (E. Langan, 2022, personal communication), so the Kandahar Province record of *O. taeniolatus* must be considered erroneous. The similar species epithets between *E. taeniolatus* and *O. taeniolatus* may have caused Wagner et al. (2016b) to mistake its identity, resulting in a *lapsus calami*. As a result, we formally remove *O. taeniolatus* from the herpetofauna of Afghanistan, Iran, and Turkmenistan. However, we note that the presence of *O. transcaspicus* **comb. et stat. nov.** in Afghanistan is still possible, particularly in northwestern provinces bordering Iran and Turkmenistan (suggested also by the SDM analysis).

## Discussion

### Distribution and conservation of *Oligodon transcaspicus* comb. et stat. nov.

The phylogenetic analysis of 12S–16S rRNA, cyt *b* fragments, morphological data and SDM mapping data support the resurrection of *Contia transcaspica* as *Oligodon transcaspicus* **comb. et stat. nov.** for Iranian and Turkmen populations previously ranked under *O. taeniolatus*. This analysis also revealed substantial genetic differentiation between populations of *O. taeniolatus* on the Indian subcontinent warranting additional revisionary work from future authors. We cannot provide a valid description for the deeply divergent lineages in Sri Lanka and mainland India due to a lack of broad morphological and genetic sampling. However, we note that the names *Oligodon fasciatus* Günther, 1864 (now considered a subspecies of *O. taeniolatus*) and *Oligodon taeniolatus* var. *ceylonicus* Wall, 1921 are both available names for Sri Lankan populations if they indeed prove to be distinct from others on the island, and/or the remainder of the Indian subcontinent. While the taxonomy of *O. taeniolatus* remains unresolved, the position of *O. transcaspicus* **comb. et stat. nov.** outside of this clade justifies its separation as a distinct species. The distribution of *O. transcaspicus* **comb. et stat. nov.** is probably more expansive than currently ascertained, and we expect that this species will eventually be found in additional localities across the Köpet–Dag Mountain region. The SDM models identified some patches of suitable habitat across the Western Caspian region and Central Iranian Desert in areas where Latifi (1991, 2000) and Safaei-Mahroo et al. (2015) reported its presence. Unfortunately, we could not trace any specimen vouchers of Latifi’s collection in the Razi Vaccine and Serum Research Institute associated with these Iranian records. Since Latifi’s specimens come from snake hunters of the institute (and were not collected by Latifi himself), and the locality data is given only at province level, these locality records should be treated with caution. Only subsequent fieldwork in these areas might verify such reports, particularly in adjacent Iranian provinces bordering Turkmenistan.

Verified records of *O. transcaspicus* **comb. et stat. nov.** also exist close to the border of Afghanistan in Mary Province, Turkmenistan. Since SDM modeling also predicts suitable habitat in this region, it is possible that this species persists in the northwestern portion of Afghanistan (specifically Herat, Badghis, Faryab and Jowzan provinces), but remains unconfirmed there due to ongoing security concerns (see Jablonski et al., 2021). The conservation of *O. transcaspicus* **comb. et stat. nov.** does not seem to be under any significant threats, however habitat degradation and human encroachment could pose a threat to some populations. Climate projections across the southern portions of Turkmenistan predict an increase in aridification and a reduction of river runoff over the following decades (Lioubimtseva, Kariyeva & Henebry, 2014; Duan et al., 2019). Because the SDM mapping analyses indicated temperature and precipitation seasonality were major factors influencing the distribution of *O. transcaspicus* **comb. et stat. nov.**, climate change could negatively impact populations. Under listings provided by the International Union for Conservation of Nature (IUCN) we would recommend classifying this species as “Least Concern”. Continued monitoring of existing populations and additional field surveys across the Köpet–Dag Mountain Range would improve our understanding of the ecology and conservation of this species.

### Taxonomic comments on the *Oligodon arnensis* species complex

Although our sampling is limited, the results from our study suggest the three-taxon statement of *O. arnensis sensu* auctorum by Bandara et al. (2022) requires additional scrutiny. Specimens we examined from Ganjam district, Odisha, India (CAS 17224–225) are well within the distribution of *O. russelius* (fide Bandara et al., 2022) but morphologically resemble *O. arnensis sensu* stricto, bearing no loreal scale, and 18–20 dark black body bands that are 2.0–3.0 dorsal scales wide. Another specimen collected near Rajamahendravaram, Andhra Province, India (CAS 94375) is close to the type locality of *O. russelius* but has 22 dorsal body bands that are 1.5–2.0 dorsal scales wide and also lacks a loreal scale, thus resembling the species diagnosis of *O. arnensis sensu* stricto. Either *O. arnensis* and *O. russelius* are sympatric in parts of eastern India, or a broad contact zone between the two species exists, which could explain why these specimens we examined have more variable color pattern conditions than previously described. There are also issues with the distribution map of *O. arnensis, O. russelius* and *O. tillacki* in Bandara et al. (2022; figure 10 therein). The map clearly separates the three taxa in India, but the figured collection localities represent a combination of specimen vouchers and observations from iNaturalist, with no meaningful way to distinguish between these lines of data. While we agree that citizen scientist databases like iNaturalist have great utility in documenting rare and understudied herpetofauna (as exemplified here by the Afghan record of *O. russelius*), they can still suffer from data quality issues (namely, limited number of visible morphological features, inaccurate locality data and misidentification errors). It is impossible to discern whether the locations in Bandara et al. (2022)’s map represent voucher specimens or iNaturalist observations, and the distribution map is not scientifically repeatable because the authors failed to provide a geolocation appendix for the iNaturalist records. Besides issues related to species distribution, we note that the p-distance (based on cyt *b*) between Sri Lankan *O. arnensis* samples and the GenBank sample Bandara et al. (2022) identified as *O*. cf. *tillacki* is only 3.6%, lower than normal p-distances separating species-level lineages of *Oligodon*. We conservatively retain the species status of *O. tillacki* because we have not directly examined the voucher specimen of this sample to verify its identity. Overall, we recommend that subsequent treatments of *O. arnensis sensu* stricto, *O. russelius*, and *O. tillacki* emphasize increased specimen and tissue collection for an integrative taxonomic approach.

Our analysis also confirmed the taxonomic identity of *O. “arnensis”* populations in northern Pakistan and Afghanistan as *O. russelius*. Nevertheless, several authors (Minton, 1966; Mertens, 1969; Khan, 2002) note the distribution of *O. “arnensis”* extends into central and southern Pakistan close to the border of western India. These locations come close to the known distribution of *O. tillacki*, especially near Gujarat State, India. However, Minton (1966) noted that all his specimens from Pakistan have black bars with white edges, a phenotypic trait that Khan (2002) also described. Kukri snakes figured by these authors show a “V-shaped” marking on the nape, although it is not a thick triangular blotch that is observed in *O. tillacki*. We believe there is little indication that *O*. “*arnensis*” populations in southern and central Pakistan represent *O. tillacki* and we maintain the identity of these populations as *O. russelius* for the time being. It is still possible that *O. tillacki* could range into this part of the country due to the continuity of habitat within this region, and we suggest future herpetological survey work and the examination of additional material to confirm this. Such a discovery could align with recent studies that have documented a biogeographic break between the right and left banks of the Indus River seen in several Pakistani amphibians and reptiles (Gowande et al., 2021; Agarwal et al., 2022; Dufresnes et al., 2022).

Additionally, our study has taxonomic implications for the recently described *O. churahensis*, as Mirza, Bhardwaj & Patel (2021) tentatively associated Pakistani samples of *O*. “*arnensis*” with this species. Like the genetic distances between *O*. cf. *tillacki* and *O. arnensis sensu* stricto, the cyt *b* p-distance between the Pakistani clade of *O. russelius* (=*O*. cf. *churahensis*) and the clade containing the type series of *O. churahensis* is low for species-level divergence, standing at only 3.3%. *Oligodon churahensis* was described by Mirza, Bhardwaj & Patel (2021) based on two specimens collected at the foothills of the western Himalayas in Himachal Pradesh, India. Bandara et al. (2022) correctly noted that literature descriptions of kukri snakes similar to *O. churahensis* were reported as *O. arnensis sensu* lato (=now *O. russelius*) by past authors (Wall, 1921; Deraniyagala, 1936; Constable, 1949; Deraniyagala, 1955). However, because Bandara et al. (2022) include data from these historical sources into their conception of *O. churahensis* (as seen in their supplementary material), diagnosing this species from *O. russelius* becomes puzzling. In the comparisons section of *O. russelius*, Bandara et al. (2022) separated this species from *O. churahensis* by the presence of 30–45 crossbars (*vs*. 48–54 in *O. churahensis*) the distance between each crossbar measured in vertebral dorsal scales (4–6 scales *vs*. 2–4 in *O. churahensis*), and by a different head shape marking (inverted Y-shaped marking *vs*. heart-shaped symbol in *O. churahensis*). However, the authors note that *O. churahensis* has 56–62 crossbars in the remaining text, especially in Table 1, where it is again compared to the 30–45 crossbars of *O. russelius*. It appears that the authors confused the true number of crossbars found in *O. churahensis* and *O. russelius*, failed to specify between “total crossbars” (including body and tail bars) and “body crossbars” (restricted from nape to vent) and did not completely summarize the color pattern traits observed in all of the sources they believe comprise *O. churahensis* (Wall, 1921; Deraniyagala, 1936; Constable, 1949; Deraniyagala, 1955; Mirza, Bhardwaj & Patel, 2021). Per the original description (Mirza, Bhardwaj & Patel, 2021), the actual number of body crossbars found in *O. churahensis* is 37–45, with the number of total crossbars noted as 48–54. Furthermore, Bandara et al. (2022) stated in their written description that *O. russelius* has 30–45 body crossbars and 6–10 tail bars. This indicates that the supposed differences in crossbar numbers are a lot less significant than previously considered. As argued by Mahony & Kamei (2021), errors and inconsistencies in taxonomic articles are inevitable, but authors should practice great care to make sure data inputted into descriptions and tables is consistent.

When we combine our own data on *O. russelius* from Afghanistan, Pakistan with the clarified characters of Bandara et al. (2022) and existing data in the literature (Wall, 1921; Constable, 1949; Brück, 1968), most color pattern and scalation traits between this taxon and *O. churahensis* overlap (Table 3). Combined with the low genetic divergence revealed by our molecular data, our study does not support the species-rank status of *O. churahensis*. It is plausible that specimens identified as *O. churahensis* represent the high-end of a geographic cline of cross-barred phenotypes found within *O. russelius* across its range, as suggested in part by Wall (1921). Alternatively, it is possible that the *O. churahensis* and *O. russelius* clades could maintain their reciprocal monophyly, albeit with low genetic divergence that would support the existence of two lineages at the subspecies level. In the absence of additional samples from the type locality of *O. churahensis* and adjacent regions of northern India and Pakistan, we believe the most appropriate decision is to relegate the species to the junior synonymy of *O. russelius*, which we do so here. A key to the *O. arnensis* species complex and related species is provided below to summarize our taxonomic changes (see Conclusions).

### Biogeographic implications

Our resurrection of *O. transcaspicus* **comb. et stat. nov.** and the clarification of *O*. “*arnensis*” and *O. taeniolatus* records from Middle and Southwest Asia (along with Pakistan) represents a small step in understanding the evolutionary history of the region’s herpetofauna. Interestingly, several Indo-Malayan reptiles found in Middle and Southwest Asia are recognized as separate taxa. Examples include *Boiga trigonata melanocephala* (Annandale, 1904), *Lycodon bicolor* (Nikolsky, 1903b) and *Ptyas mucosa nigriceps* Terentjev & Chernov, 1949 (Orlov et al., 2018; Amarasinghe et al., 2023). Other species found in Middle/Southwest Asia whose close relatives originate from the Indo-Malayan realm include *Naja oxiana* Eichwald, 1831 and *Eublepharis macularius* Blyth, 1854b. For the latter species (*N. oxiana* and *E. macularius*), phylogeographic studies show there is low genetic differentiation between populations opposite of the Hindu Kush, implying that these species likely experienced recent and rapid range expansions into Middle/Southwest Asia (Kazemi et al., 2021; Agarwal et al., 2022). The uniqueness of the evolutionary lineage representing *O. transcaspicus* **comb. et stat. nov.** paints a different picture, because the presented molecular, morphological and SDM evidence implies that the divergence of its common ancestor into present-day Iran and Turkmenistan occurred much earlier. Though it is difficult to hypothesize the exact timing of divergence for *O. transcaspicus* **comb. et stat. nov.**, the high topography surrounding the Hindu Kush and the arid Registan-North Pakistan sandy deserts to its south probably reduced opportunities for range expansion to periods in the late Miocene and the Plio-Pleistocene, when climatic conditions promoted the growth of more suitable semi-arid steppes and shrublands (Hoorn, Ohja & Quade, 2000; Merceron et al., 2004; Modarres et al., 2019). The diversity and cladogenesis of Indo-Malayan-affiliated elements of Middle and Southwest Asia’s amphibians and reptiles remain poorly understood, presenting intriguing questions for future research. The acquisition of new material along with more comprehensive phylogeographic analyses will undoubtedly shed light on these unique animals and their taxonomic and evolutionary status.

## Conclusions

We carefully reviewed the status of the two kukri snake species found in Middle and Southwestern Asia, the banded kukri snake *O. “arnensis”* and the streaked kukri snake *O. taeniolatus*, leading to several taxonomic and distributional clarifications. For *O. taeniolatus*, we found that specimens from the Köpet-Dag Mountain Range of northern Iran and southern Turkmenistan were phylogenetically nested in a different intrageneric grouping compared to nominotypical *O. taeniolatus* on the Indian subcontinent. To fix the paraphyly recovered by these analyses, we resurrected the junior synonym *Contia transcaspica* and provided a thorough morphological redescription supplemented with SDM mapping to explore its potential range. We also found that Afghanistan and northern Pakistan records of kukri snakes previously recognized as *O. arnensis* should in fact be assigned to the species *O. russelius*. This latter species forms a sister clade to the recently described *O. churahensis* from northern India, but is only separated by a small genetic divergence of 3.3% based on cytochrome *b*. Because the morphology between *O. russelius* and *O. churahensis* overlaps, we consider both species to be conspecific, and relegate *O. churahensis* to the junior synonymy of *O. russelius*. Our investigation further revealed that there are no records of *O. taeniolatus* from Afghanistan, as the two past reports from the literature refer to a misidentified specimen of *O. russelius*, and a misidentified scincid lizard, respectively. As a consequence, we remove *O. taeniolatus* from the snake fauna of Afghanistan, Iran, and Turkmenistan. The two kukri snakes found in Middle and Southwestern Asia are *O. transcaspicus* **comb. et stat. nov.** and *O. russelius*. These two species represent key Indo-Malayan herpetofaunal elements in a region that primarily consists of Palearctic amphibians and reptiles. Their ranges in Middle and Southwest Asia appear to be influenced by past climate change across the Hindu Kush Mountain range, in addition to past biotic and abiotic fluctuations that occurred during the Plio-Pleistocene. Future studies that explore the biogeographic origins of these animals are recommended. We also suggest that additional taxonomic work on *O. arnensis* and *O. taeniolatus* is needed for the Indian subcontinent populations. The number of recognized species in *Oligodon* remains at 88 (fide Yushchenko et al., 2023a, 2023b).

### Key to members of the *Oligodon arnensis* species complex and related species

We provide a new key summarizing the taxonomic changes of our study. Our phylogenetic results separate *O. arnensis sensu* auctorum and *O. taeniolatus sensu* auctorum into two separate clades, however we note that Smith (1943) originally included both taxa in the same morphological species grouping. We tentatively follow Smith’s classification for this key and include *Oligodon affinis* Günther, 1862, *Oligodon brevicauda* Günther, 1862, *O. calamarius*, *Oligodon erythrorhachis* Wall, 1910, *O. sublineatus* and *O. taeniolatus*, in addition to members currently assigned to the *O. arnensis* species complex, acknowledging that this does not reflect the phylogenetic results of our study. Data used to create this key originate from Wall (1923), Smith (1943), Bandara et al. (2022), Das et al. (2022) and our own examined material.

1a. Anterior dorsal scale rows in 15 rows (2)

1b. Anterior dorsal scale rows in 17 rows (7)

2a. Internasals and prefrontals fused, loreal absent, dorsal color pattern red in life consisting of reddish brown and dark brown longitudinal stripes with occasional interspaced spots (*Oligodon brevicauda*)

2b. Internasals and prefrontals separate, loreal condition variable, dorsal pattern not consisting of longitudinal series of stripes (3)

3a. 5–6 supralabials with only the 3^rd^ supralabial in contact with the orbit, narrow contact between the 4^th^ and 5^th^ supralabial, usually three posterior temporal scales, restricted to the Köpet-Dag Mountain range of Iran and Turkmenistan (*Oligodon transcaspicus* **comb. et stat. nov.**)

3b. More than six supralabials with more than one scale contacting the orbit and broad contact between all supralabials (4)

4a. Loreal present, dorsal color pattern variable but always without a red vertebral stripe and lacking narrow black crossbars (5)

4b. Loreal absent, dorsal color pattern dark gray consisting of a bright red vertebral stripe and approximately 29 black, narrow crossbars on the body, seven on the tail, all crossbars black with white edges. Known only from northeast India (*Oligodon erythrorhachis*)

5a. Usually more than 160 ventral scales, color pattern with conspicuous broad crossbars or blotches that maintain the same size across the body, a narrow vertebral stripe normally present, two-fifths of the retracted hemipenis bilobed and spinose (*Oligodon taeniolatus*)

5b. Usually less than 160 ventral scales, color pattern consisting of irregularly shaped crossbars that mostly concentrated on the anterior half of body and decrease in size posteriorly, retracted hemipenis unilobed or only bilobed at the apex (6)

6a. Light vertebral stripe present, crossbars dark brown and narrow, concentrated on the anterior half of the body, ventral surface with separate equally arranged rectangular spots, retracted hemipenis unilobed (*Oligodon calamarius*)

6b. Light vertebral stripe absent, crossbars broad and irregular, restricted to the anterior third of the body, ventral surface with longitudinal rows of spots forming three distinct rows of stripes, retracted hemipenis bilobed at apex (*Oligodon sublineatus*)

7a. Less than 160 ventrals, dorsum grayish brown to brown with small reticulations, crossbars present or absent (8)

7b. More than 160 ventrals, dorsum olive brown with dark crossbars (9)

8a. Ventral surface white below with dark square spots, dorsal scales 17 at midbody, 129–145 ventrals (*Oligodon affinis)*

8b. Ventral surface distinctively gray blue in life, dorsal scales 15 at midbody, 144–159 ventrals (*Oligodon melaneus*)

9a. Loreal always absent, 164–188 ventrals in both sexes, 15–20 dark body crossbars that are 2–3 vertebral dorsal scales wide, 7–12 vertebral dorsal scales between each crossbar (*Oligodon arnensis*)

9b. Loreal present, 169–207 ventrals in both sexes, 30–54 dark body crossbars that are less than 2 vertebral dorsal scales wide, 3–6 vertebral dorsal scales between each crossbar (*Oligodon russelius*)

9c. Loreal present (rarely absent), 180–201 ventrals in both sexes, 25–35 dark body crossbars with the first 5–8 distinctly enlarged, 4–6 vertebral dorsal scales wide and 2–4 vertebral dorsal scales between each crossbar (*Oligodon tillacki*).

## Supplemental Information

10.7717/peerj.15185/supp-1 Supplemental Information 1 Other specimens of *Oligodon* examined, organized based on their morphological identification based on Bandara et al. (2022).

10.7717/peerj.15185/supp-2 Supplemental Information 2 Supplementary Tables.

## Additional Information and Declarations

### Field Study Permissions

The following information was supplied relating to field study approvals (*i.e*., approving body and any reference numbers): Field works, including collection of the samples and animals in the field, was performed outside of any protected area, in the framework of a project contract signed by International Center for Science, High Technology and Environmental Sciences, Kerman, Iran (contract number 1.87, issued at 11.04.2008). The contract bears a permission to collect the reptile samples outside of any protected area of Department of the Environment (specified in www.doe.ir) that needed extra permissions. Specimen collection protocols and animal operations followed the Institutional Ethical Committee of International Center for Science, High Technology and Environmental Sciences, Kerman, Iran (certificate number 1.87-1).

### DNA Deposition

The following information was supplied regarding the deposition of DNA sequences: The sequences of 12S–16S rRNA and cyt b genes are available at GenBank: OQ092426; OQ099833–OQ099837; and OQ116816–OQ116825 (Table 1).

### Data Availability

The following information was supplied regarding data availability: Specimens examined in this study are deposited in herpetological collections of the following museums: 1. California Academy of Sciences, San Francisco, USA (CAS): *Oligodon arnensis*: CAS 17224–25, CAS 1722; *Oligodon russelius*: CAS 94375; *Oligodon transcaspicus*: CAS 180042. 2. Comenius University Herpetological Collection, Bratislava, Slovakia (CUHC): *Oligodon russelius*: CUHC 7904. 3. Department of Zoology, Moravian Museum, Brno, Czech Republic (MMB): *Oligodon russelius*: MMB 28497. 4. National Museum of Natural History, Washington, DC, USA (USNM): *Oligodon arnensis*: USNM 254625, USNM 254655; *Oligodon russelius*: USNM 267008. 5. Zoological Institute of Russian Academy of Sciences, St. Petersburg, Russia (ZISP): *Oligodon transcaspicus*: ZISP 9868, ZISP 18334, ZISP 18976. 6. Zoological Museum of Moscow State University, Moscow, Russia (ZMMU): *Oligodon transcaspicus*: ZMMU Re-5589, ZMMU Re-6155, ZMMU Re-16687.
