# Supplementary material for: Kukri snakes Oligodon Fitzinger, 1826 of the Western Palearctic with the resurrection of Contia transcaspica Nikolsky, 1902 (Reptilia, Squamata, Colubridae)
Source: PeerJ. 2023 May 18;11:e15185. doi: 10.7717/peerj.15185 (PMC10200101; doi:10.7717/peerj.15185)
Supplement: Supplemental Information 1 [file peerj-11-15185-s001.docx]

**Supplementary Material 1.** Other Specimens of *Oligodon* examined, organized based on their morphological identification based on Bandara et al. (2022).

*Oligodon arnensis* (N=5). **India.** CAS 17224–25 from “Ganjam” Odisha; CAS 17226 from Tamil Nadu; — **Sri Lanka.** USNM 254625 from “Marawila” Puttalam District, North Western Province; USNM 254655 from “Mundel, 3 mi from, Nawadamkulama”, Puttalam Distrct, North Western Province.

*Oligodon russelius* (N=2). **India.** CAS 94375 from “15 mi SW of Rajah Mundry (Stop 158)” [24.1 km SW of Rajamahendravaram, Andhra Pradesh (16.8650°N, 81.6631°E); — **Nepal.** USNM 267008 from Smithsonian Institution Camp, vicinity of Sauraha, Royal Chitwan National Park, Chitwan District, Bagmati Province.
