# Supplementary material for: Kukri snakes Oligodon Fitzinger, 1826 of the Western Palearctic with the resurrection of Contia transcaspica Nikolsky, 1902 (Reptilia, Squamata, Colubridae)
Source: PeerJ. 2023 May 18;11:e15185. doi: 10.7717/peerj.15185 (PMC10200101; doi:10.7717/peerj.15185)
Supplement: Supplemental Information 2 [file peerj-11-15185-s002.docx]

**Supplementary Table 1.** Tabled list of localities of *Oligodon transcaspicus* **comb. et stat. nov.** with latitude and longitude coordinates used for species distribution modelling.

| **No.** | **Species** | **Source** | **Country** | **Locality** | **Coordinates** |
| --- | --- | --- | --- | --- | --- |
| **1** | *O. transcaspicus* | Orlov et al., 2018 | Turkmenistan | Danata spring, Kyurendag | 39.0916ºN, 55.1611ºE |
| **2** | *O. transcaspicus* | Orlov et al., 2018 | Turkmenistan | Yoldere Gorge | 38.5125ºN, 56.3869ºE |
| **3** | *O. transcaspicus* | Orlov et al., 2018 | Turkmenistan | Akderekjhan spring, north of Kara-Kola | 38.2977ºN, 56.3272ºE |
| **4** | *O. transcaspicus* | Orlov et al., 2018 | Turkmenistan | Chandyr Valley | 38.1500ºN, 56.0166ºE |
| **5** | *O. transcaspicus* | Orlov et al., 2018 | Turkmenistan | Makhtum–Kala | 38.4160ºN, 56.4263ºE |
| **6** | *O. transcaspicus* | Orlov et al., 2018 | Turkmenistan | Kara–Yalchi Gorge | 38.4500ºN, 57.2000ºE |
| **7** | *O. transcaspicus* | Orlov et al., 2018 | Turkmenistan | Eishem (Egshem) spring | 38.9416ºN, 56.7500ºE |
| **8** | *O. transcaspicus* | Orlov et al., 2018 | Turkmenistan | Aidere River Valley, Kurygol | 38.4038ºN, 56.7569ºE |
| **9** | *O. transcaspicus* | Orlov et al., 2018 | Turkmenistan | Arvaz Rivery Valley | 38.3500ºN, 57.0833ºE |
| **10** | *O. transcaspicus* | Orlov et al., 2018 | Turkmenistan | 7 km north of Saivan | 38.5733ºN, 56.7400ºE |
| **11** | *O. transcaspicus* | Orlov et al., 2018 | Turkmenistan | between Firyuza and Chuli | 37.9427ºN, 58.0611ºE |
| **12** | *O. transcaspicus* | Orlov et al., 2018 | Turkmenistan | Shamli | 37.7166ºN, 58.6916ºE |
| **13** | *O. transcaspicus* | Orlov et al., 2018 | Turkmenistan | Babazo Gorge | 37.7500ºN, 58.3250ºE |
| **14** | *O. transcaspicus* | Orlov et al., 2018 | Turkmenistan | Qaranqi Gorges | 37.7833ºN, 58.2833ºE |
| **15** | *O. transcaspicus* | Orlov et al., 2018 | Turkmenistan | Dana–Germab spring | 35.9385ºN, 61.2129ºE |
| **16** | *O. transcaspicus* | Orlov et al., 2018 | Turkmenistan | Nardyvanly spring | 35.7750ºN, 61.3746ºE |
| **17** | *O. transcaspicus* | *this paper* (A. V. Pavlenko, pers. comm.) | Turkmenistan | Pordere, Sumbar Valley | 38.2333ºN, 56.9750ºE |
| **18** | *O. transcaspicus* | *This paper* (A. V. Pavlenko, pers. comm.) | Turkmenistan | Seqiz-Khan Gorge | 38.9166ºN, 56.1166ºE |
| **19** | *O. transcaspicus* | Dotsenko, 1984 | Turkmenistan | Kara–Kala environs | 38.4578ºN, 56.3105ºE |
| **20** | *O. transcaspicus* | CAS HERP 180042 | Turkmenistan | Goalon (Goudan) | 37.6301ºN, 58.4075ºE |
| **21** | *O. transcaspicus* | Latifi, 2000; this paper | Iran | Dashli Borun | 37.6367ºN, 54.8171ºE |
| **22** | *O. transcaspicus* | *this paper* | Iran | Bazangan Lake | 36.3105ºN, 60.4809ºE |
| **23** | *O. transcaspicus* | *this paper* | Iran | 5 km southwest of Mashhad | 36.1866ºN, 59.5088ºE |

**Supplementary Table 2.** Primers used in this study

| **Gene** | **Primer name** | **Sequence (5’–3’)** | **Reference** |
| --- | --- | --- | --- |
| 12S–16S | Oligo12S2LM | ACACACCGCCCGTCACCCT | Green et al., 2010 |
| 12S–16S | Oligo16S5H | CTACCTTTGCACGGTTAGGATACCGCGGC | Green et al., 2010 |
| 16S | Oligo16S1LM | CCGACTGTTGACCAAAAACAT | Green et al., 2010 |
| 16S | 16S-H-1 | CTCCGGTCTGAACTCAGATCACGTAGG | Hedges, 1994 |
| cytochrome b | H14910 | GACCTGTGATMTGAAAAACCAYCGTT | Chen et al., 2014 |
| cytochrome b | THRSN2 | CTTTGGTTTACAAGAACAATGCTTTA | Chen et al., 2014 |
